# Cytotoxin-mediated silk gland organ dysfunction diverts resources to enhance silkworm fecundity by potentiating nutrient-sensing IIS/TOR pathways

**DOI:** 10.1016/j.isci.2024.108853

**Published:** 2024-01-11

**Authors:** Ping Ying Lye, Chika Shiraki, Yuta Fukushima, Keiko Takaki, Mervyn Wing On Liew, Masafumi Yamamoto, Keiji Wakabayashi, Hajime Mori, Eiji Kotani

**Affiliations:** 1 Department of Applied Biology, Kyoto Institute of Technology, Sakyo-ku, Kyoto 606-8585, Japan; 2 Biomedical Research Center, Kyoto Institute of Technology, Sakyo-ku, Kyoto 606-8585, Japan; 3 Institute for Research in Molecular Medicine, Universiti Sains Malaysia, Minden, Penang 11800, Malaysia; 4 ICLAS Monitoring Center, Central Institute for Experimental Animals, 3-25-12 Tonomachi, Kawasaki-ku, Kawasaki 210-0821, Japan; 5 Graduate Division of Nutritional and Environmental Sciences, University of Shizuoka, 52-1 Yada, Suruga-ku, Shizuoka 422-8526, Japan

**Keywords:** Biological sciences, Molecular biology, Bioengineering

## Abstract

Energy reserves, primarily stored in the insect’s fat body, are essential for physiological processes such as reproduction and cocoon formation. However, whether these processes are mutually constraining is unknown. Here, we showed that cocoon-free silkworms accumulate amino acid constituents of silk proteins in the hemolymph and maintain lipid and sugar reserves in the pupal fat body by repressing the expression of sericin and fibroin genes in the middle and posterior silk glands, respectively, via butterfly pierisin-1A catalytic domain expression. This, in turn, upregulates insulin/insulin-like signaling and target of rapamycin (IIS/TOR) signaling, which enhances vitellogenesis and accelerates ovarian development, thus contributing to increased fecundity. The impacts of semi-starvation on fecundity and egg hatchability were also less pronounced in cocoon-free silkworms compared with wildtype silkworms. These data uncover the resource allocation trade-off between cocoon formation and fecundity and demonstrate that nutritional signaling plays a role in regulating silkworm reproduction.

## Highlights

• Repressing silk protein synthesis inhibits cocooning and increases final body size
• Excess hemolymph amino acids and high energy reserves upregulate IIS/TOR signaling
• TOR regulates vitellogenin expression at transcriptional and translational levels
• Nutritional status strongly influences reproductive outcomes in silkworms

## Graphical abstract

**Figure:**
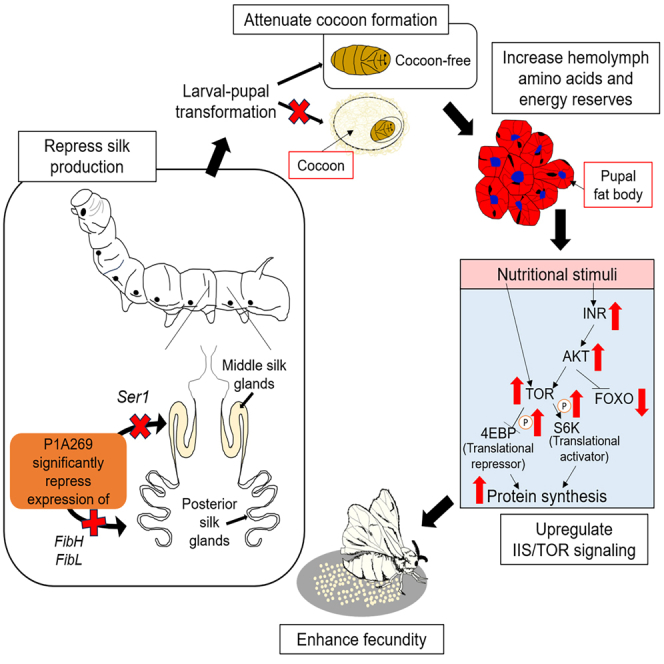

## Introduction

Cocoon formation, which is present across the diverse insect taxa, primarily serves as a protective barrier during the insect pupal stage, when they are immobile, shielding them from predators, parasites, and harsh environments.^1^ The most extensively studied example is the cocoon of silkworms (*Bombyx mori*), which is composed of silk.^2^ The economic significance of silk in international trade has been the primary driving factor behind the domestication of silkworms from their wild counterpart (*Bombyx mandarina*), which may date back to more than 5000 years^3^^,^^4^ Human-directed selection of silkworms to enhance the silk yield through the production of larger cocoons during domestication requires a substantial investment of energy resources, as evidenced by the enrichment of genes that are related to the ribosome biogenesis pathway^5^ and nitrogen and carbon metabolism^6^ in the silk glands, in comparison to that of *B. mandarina*.

In insects, including silkworms, the fat body plays a vital role as a central energy storage depot which facilitates the energy mobilization to meet the metabolic demands during the various physiological processes such as starvation, larval-pupal metamorphosis, reproduction, immune responses, and adult flight.^7^^,^^8^^,^^9^ However, because of the limited resources within the fat body and competition for resource allocation, trade-offs inevitably occur. Insect reproduction,^10^ similar to cocoon formation, is an energy expensive process. Reproductive fitness, that is measured in terms of fecundity, is crucial to ensure the survival and persistence of the species. However, potential consequences of the high energy investment in silk production, as a result of selective breeding, on their reproductive fitness remain poorly understood.

Silkworm cocoons consist of two silk proteins, sericin (Ser1 and Ser3) and fibroin (FibH and FibL), that are synthesized in the middle silk glands (MSGs) and posterior silk glands (PSGs), respectively. The manipulation of the silk glands through genetic or surgical approaches is essential to elucidate the relationship between silk production and fecundity in silkworms. However, silkworms completely lacking cocooning ability without adverse effects have yet to be discovered. The Naked pupa (*Nd*), which is a naturally occurring silkworm mutant, despite the strain name, still produces a loose cocoon that mainly contains the protein sericin rather than completely inhibiting silk production.^11^^,^^12^ The surgical removal of silk glands to inhibit the cocoon formation has resulted in pupation failure.^13^ To date, there have also been no reported successful genetic modifications in silkworms to repress silk production without inducing physiological defects. Several studies have utilized the TALEN and CRISPR techniques to knockout sericin or fibroin genes in the silk glands, which enable larval growth, but they unfortunately lead to varying levels of pupation failure or pupal lethality.^14^^,^^15^

Pierisin-1, derived from the cabbage butterfly (*Pieris rapae*), is a potent cytotoxin protein with DNA mono(ADP-ribosyl)ation (MARylation) activity and its expression in living cells is impractical due to its cytotoxic effect.^16^^,^^17^ However, pierisin-1A (P1A), a homolog of pierisin-1, exhibits only 5% of the DNA MARylation activity and can repress fibroin gene expression without inducing cell death.^18^ In order to investigate the consequences of increased investment in silk production on the reproductive fitness of silkworms, we generated silkworms devoid of cocoon formation capability by targeting the expression of the catalytic domain of P1A, which lacks the secretory signal and putative receptor binding-domain sequences (2–269 amino acids; P1A269) in the MSGs and PSGs. We found that repression of the expression of sericin and fibroin genes has notable effects on silkworm final body mass, nutrient metabolism and reproductive output. This research reveals that reallocating resources away from cocoon formation may be associated with the enhancement of reproductive outcomes in insects.

## Results

### Establishment of the cocoon-free silkworms by P1A269-mediated repression of sericin and fibroin gene expression in the silk glands

We first generated a transgenic silkworm line expressing the P1A269 transgene under the control of the sericin (*Ser1*) promoter in the MSGs (Figure S1), named the Ser1-free cocoon silkworm line. SDS-PAGE analysis showed that the fibroin (FibH and FibL) proteins were primarily observed in the Ser1-free cocoons, and the Ser1 protein detected in the wildtype *w1-pnd* (WT) and fibroin-free cocoons^18^ was not observed (Figure S2). While an approximately 200 kDa band, which is similar to the reported molecular weight of the Ser3 protein,^19^^,^^20^ was observed (Figure S2, asterisk∗). LC-MS/MS analysis of the excised gel band detected partially fragmented FibH proteins in addition to the Ser3 protein (data not shown). In view that Ser1 is the major sericin protein in cocoons and is highly expressed in comparison to Ser3 in the MSGs during normal silk production,^20^ the Ser1-free cocoon silkworms likely produce cocoons consisting mainly of fibroin with a negligible amount of sericin.

By crossing this line with the fibroin-free cocoon silkworm line,^18^ we successfully generated a novel transgenic line devoid of cocoon formation ability, with which the expression of sericin and fibroin genes were repressed, known as the cocoon-free silkworm line (See STAR Methods). The cocoon-free silkworm progeny exhibited both red- and green-fluorescing eyes and nerve cord (Figure S3), and the expression of P1A269 in the MSGs (Figure S4) was confirmed by immunoblot analysis with an anti-FLAG tagged antibody.

### Repression of silk protein synthesis resulted in amino acid accumulation in the larval hemolymph without physiological defects

The mRNA expression levels of sericin and fibroin genes in silk glands of WT (serving as controls) and cocoon-free silkworms were evaluated by quantitative RT-PCR (qRT-PCR). The 18s ribosomal RNA (*18S rRNA*) was used as a reference gene for normalization, given its comparable levels in the silk glands between WT and cocoon-free silkworms at the specific fifth-instar larval stage (Figure S5). We observed a significant reduction in the expression levels of the primary genes that encode for sericin (*Ser1*) and fibroin (*FibH* and *FibL*) proteins in the cocoon-free silkworm larval silk glands in comparison to the WT silkworms. The *Ser1* mRNA levels in the MSGs of cocoon-free silkworms were substantially reduced by 97% (p < 0.001; Figure S6A) compared to the WT silkworms. Similarly, the mRNA levels of *Ser2* (reduced by 39%, p < 0.05; Figure S6B) and *Ser3* (reduced by 45%, p < 0.01; Figure S6C) also reduced, but to a lesser extent than *Ser1*. These differences might be attributed to the region-specific expression patterns of the sericin (*Ser1, Ser2*, and *Ser3*) genes in the MSGs of silkworms.^19^^,^^20^ Ser2 detected in anterior region of the MSGs is known as the larval silk; while Ser3, also produced in the anterior region, is less abundant relative to Ser1 in cocoons.^20^ Therefore, we infer that P1A269 expression under Ser1 promoter control more strongly repressed the Ser1, which is highly expressed in the middle and posterior regions of the MSGs and identified as the major sericin protein in cocoons, compared to Ser2 and Ser3. The *FibH* and *FibL* mRNA levels in the PSGs of the cocoon-free silkworm larvae were also substantially reduced by 98% (p < 0.01; Figure S6D) and 88% (p < 0.001; Figure S6E), respectively, compared to WT silkworms.

**Figure 1:**
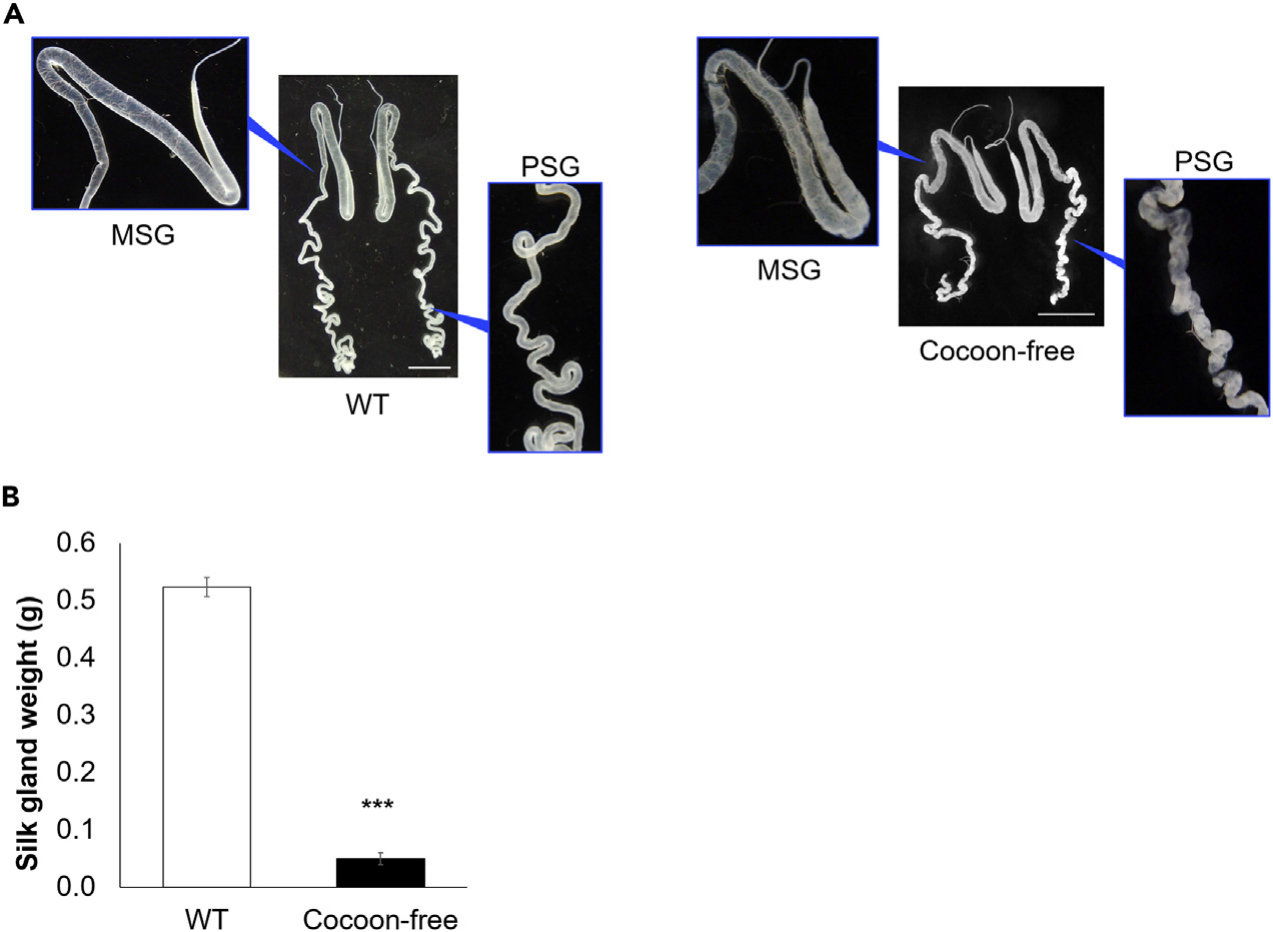
Repression of silk protein synthesis resulted in silk gland morphological abnormalities and weight reduction (A) Representative images of the silk glands sampled from day 6 fifth instar larvae of the WT and cocoon-free silkworms are shown. The enlarged images of the cocoon-free silkworm larvae show the structural deformities in the middle silk glands (MSGs) and posterior silk glands (PSGs). (Scale bar, 1 cm). (B) Average weight of the silk glands from day 6 fifth instar larvae of WT and cocoon-free silkworms (n = 7). Data represent mean ± SEM, ∗∗∗p < 0.001 according to the Student’s *t* test.

Moreover, the expression of P1A269 resulted in morphological abnormalities (Figure 1A) as well as a substantial decrease in the larval silk gland weight (Figure 1B). Specifically, the cocoon-free silkworm larvae showed distorted and shortened PSGs, and dilated anterior and posterior MSGs (Figure 1A); however, neither DAPI staining (Figure S7) nor DNA fragmentation analysis (Figure S8) revealed signs of programmed cell death (PCD) in the MSGs and PSGs. The nuclei of ASGs, MSGs and PSGs in cocoon-free silkworm larvae displayed filamentous morphology similar to that of WT silkworms (Figure S7A). Furthermore, nuclear condensation and DNA fragmentation,^21^ typically associated with PCD observed at the pupal stage (Figures S7B and S8), were not observed in the silk glands of WT and cocoon-free silkworm larvae (Figures S7A and S8). The silk glands of the cocoon-free silkworm larvae weighed 91% less than those of the WT silkworm larvae (p < 0.001; Figure 1B). Repression of silk protein synthesis in the silk glands resulted in the loss of cocoon formation ability in cocoon-free silkworms (Video S1); however, the growth and development of cocoon-free silkworms were unaffected. No discernible differences were observed in the duration of progression from larvae to pupation and the timing of molting during each instar stage between WT and cocoon-free silkworms (Figure S9). The cocoon-free silkworm larvae also exhibited similar cocoon-spinning behavior as the WT silkworms (Video S2).

Video S1. Larval-pupal metamorphic transition, related to Figure 1 The left and right silkworms are from the WT and cocoon-free lines, respectively.

Video S2. Silkworms spin cocoons in a figure-of-eight head pattern, related to Figure 1 The left and right silkworms are from the WT and cocoon-free lines, respectively.

According to previous reports,^13^^,^^22^^,^^23^^,^^24^ the suppression of cocoon formation leads to the accumulation of amino acids that are the building blocks of silk proteins in the silkworm hemolymph. Indeed, we compared the concentrations of amino acids in larval hemolymph after gut purge and observed elevated levels of serine, glycine, alanine and tyrosine, which are the major constituents of silk proteins,^22^^,^^25^ in cocoon-free silkworms compared to the WT silkworms (Table 1; Figure S10). Conversely, lower levels of histidine were noted in the larval hemolymph of cocoon-free silkworms compared to the WT silkworms (Table 1; Figure S10). This difference in histidine levels may be attributed to the increased silk gland cell volume during silk protein synthesis in WT silkworms, resulting in increased histidine release during metamorphic tissue remodeling,^23^^,^^26^^,^^27^ a process which is not present in the cocoon-free silkworms. Interestingly, there was no arrest in the larval-pupal metamorphosis (Video S1) despite the accumulation of silk protein amino acid constituents in the cocoon-free silkworm larval hemolymph, which contradicts previous findings.^13^ The survival rate of late fifth instar larvae to the adult stage was comparable between the WT (∼98%) and cocoon-free (∼96%) silkworms. There was no intentional selection for specific individuals during the establishment of the cocoon-free silkworm line. The cocoon-free silkworm line was maintained stably in the laboratory for over 20 generations without any discernible physiological deficiencies. Collectively, the data indicate that the P1A269-mediated repression of silk production and the amino acid accumulation in the larval hemolymph, resulting from the inhibition of cocoon formation, did not affect the development or viability of pupae.

**Table 1:** Repression of silk protein synthesis led to the accumulation of amino acids in cocoon-free silkworms

Amino acid	WT (nmol/mL)^c^	Cocoon-free (nmol/mL)^c^
Sericin^a^	4010 ± 102	19126 ± 238^∗∗∗^
Glycine^a^	3217 ± 85	67270 ± 2783^∗∗^
Tyrosine^a^	2107 ± 96	3470 ± 38^∗^
Alanine^a^	733 ± 82	1717 ± 65^∗^
Histidine^b^	23311 ± 1325	11857 ± 176^∗^

The concentrations (in nmol/mL) of the amino acid related to tissue remodeling and major amino acid constituents of silk proteins in hemolymph of WT and cocoon-free silkworm larvae are presented. (Complete data on amino acid concentrations in the hemolymph of WT and cocoon-free silkworm larvae is shown in Figure S10).

a Major amino acid constituents of silk proteins.

b Amino acid related to tissue remodeling upon metamorphosis.

c Data represent mean ± SEM (n = 3); ∗p < 0.05; ∗∗p < 0.01; ∗∗∗p < 0.001 according to the Student’s *t* test.

### Repression of silk protein synthesis enhanced the pupal size and energy reserves

**Figure 2:**
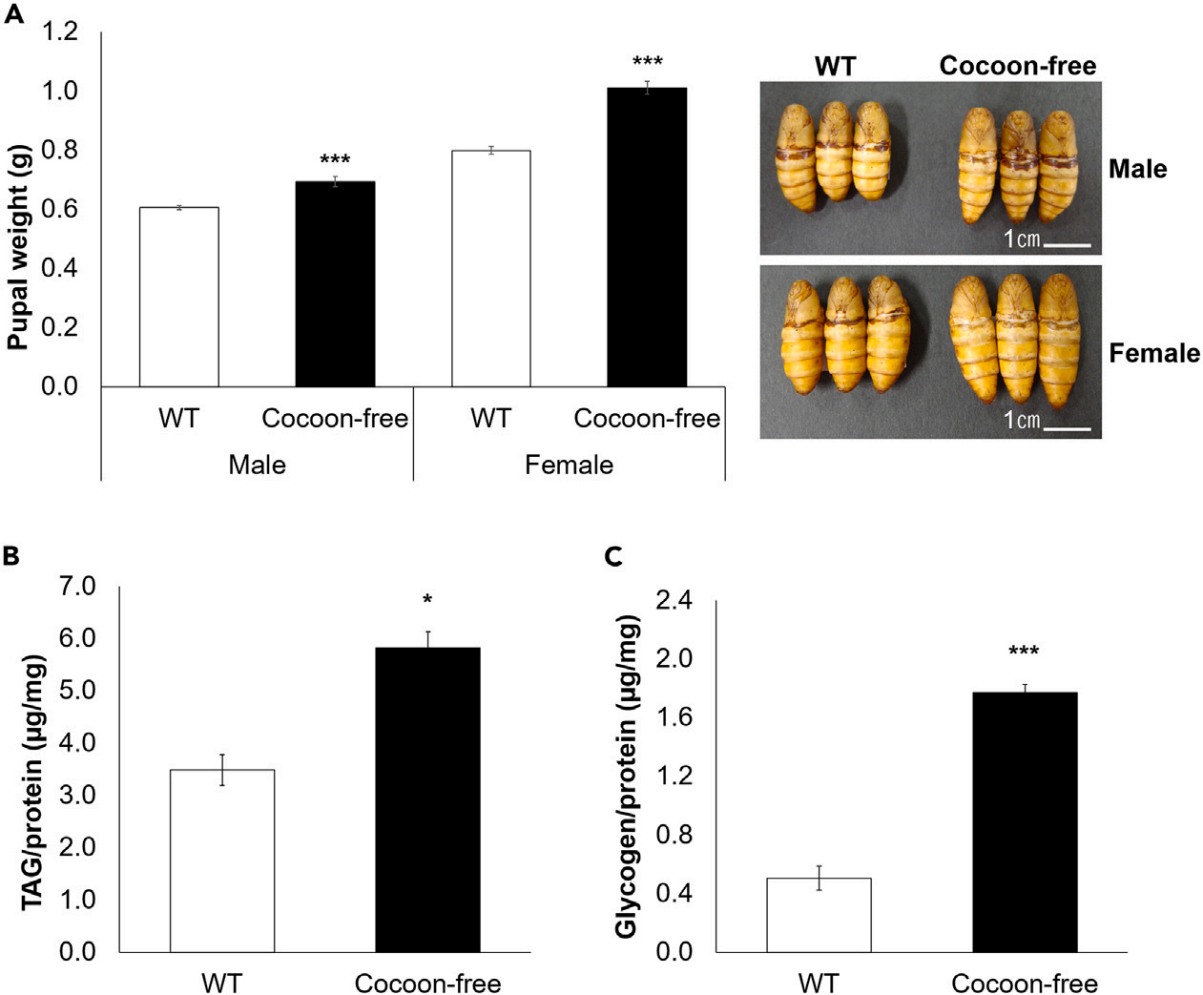
Repression of silk protein synthesis reduced the weight loss during larval-pupal metamorphosis and enhanced the energy reserves in the cocoon-free silkworm pupae (A) Average weight of the WT and cocoon-free silkworm pupae of both sexes (n = 16). Representative images of male and female WT and cocoon-free silkworm at day 0 post-pupation (pp) are shown (n = 3). (Scale bar, 1 cm). (B) Triacylglyceride (TAG) and (C) glycogen levels in the pupal fat bodies of female WT and cocoon-free silkworms. The y axis represents metabolite levels normalized to the total soluble protein concentration in the fat bodies (n = 3). Data represent mean ± SEM; ∗p < 0.05; ∗∗∗p < 0.001 according to the Student’s *t* test.

Because the pupae and adults do not feed, the final mass of last larval instar determines the final body size of various insects including the silkworms.^28^ To investigate the influence of repressing silk protein synthesis on the final body size, we reared WT and cocoon-free silkworms under the same environmental conditions. The larvae were reared at a density of 60 per 135 cm^2^ case and were allowed to feed *ad libitum*, starting from the fourth instar (See STAR Methods). The results revealed that both male and female cocoon-free silkworm larvae metamorphosed into pupae with notably larger body sizes compared to those of the WT silkworms (Figure 2A). Specifically, the WT silkworms experienced a substantial weight loss during larval-pupal metamorphosis, with the males losing ∼35% and the females losing ∼33% of their initial weight, whereas the cocoon-free silkworms only lost ∼20% and ∼16% of their initial weight in the males and females, respectively (p < 0.001; Figure S11A). These results demonstrate that the cocoon-free silkworms retained a higher proportion of their prepupal weight. Notably, the difference in the pupal weight between the WT and cocoon-free silkworms was more pronounced in the females (∼21%) than in the males (∼12%) (Figure 2A). Dry weight is often used as an indicator of nutrient content. We observed that both male and female cocoon-free silkworm pupae retained a higher amount of water compared to the WT silkworms (1.3-fold increase, p < 0.001; Figure S11B); however, after lyophilization through freeze drying, the cocoon-free silkworm pupae had a significantly greater pupal dry weight than that of the WT silkworms of both sexes (1.4-fold increase, p < 0.001; Figure S11C). The results demonstrate that although the cocoon-free silkworm pupae retained more water, they also possessed a higher concentration of dry matter, which suggests a potential alteration in the nutritional composition. These findings indicate that repression of silk protein synthesis has an obvious impact on the silkworm body conditions, with a greater effect observed in females than in males.

Insects store energy reserves in the form of triacylglyceride (TAG) and glycogen in the fat body.^7^ These reserves are mobilized to meet the energy requirements of the various tissues,^7^ including the intensive silk protein synthesis in the silk glands during the late fifth instar larval stage.^29^ To investigate how repressing silk protein synthesis affects the energy reserves, we analyzed the TAG and glycogen levels in the pupal fat bodies of the female WT and cocoon-free silkworms that were fed *ad libitum* as larvae. We found a significant difference in the TAG levels between the WT and cocoon-free silkworms, with the latter showing a 1.7-fold increase in lipid accumulation (p < 0.05; Figure 2B). The glycogen levels in the cocoon-free silkworms followed a similar trend, with a substantial 3.5-fold increase compared with the WT silkworms (p < 0.001; Figure 2C). Interestingly, while the TAG concentrations were higher than glycogen concentrations in the pupal fat bodies of female cocoon-free silkworms, the amount of increase in glycogen content exceeded TAG when cocoon formation was inhibited. These results suggest that repressing silk protein synthesis had a more pronounced impact on sugar storage compared to lipid storage in the fat body of silkworms.

### Increased energy reserves have implications on the silkworm reproduction

In silkworms, ovarian development begins during the pupal stage,^30^ a period when the feeding activity ceases.^31^ Furthermore, extensive production of yolk proteins during the insect reproductive process can be energetically expensive, necessitating mobilization of energy reserves from the fat body.^10^ Thus, we assessed the influence of changes in fat body energy reserves on the silkworm reproduction by determining the egg production (fecundity) and hatchability of laid eggs. Specifically, we examined whether fecundity and egg hatchability were affected in the adult female WT and cocoon-free silkworms when the larvae were fed *ad libitum* or semi-starved. The semi-starved group comprised larvae reared at a density 2-fold higher than that of the *ad libitum* group, with limited food availability (see STAR Methods for further information).

**Figure 3:**
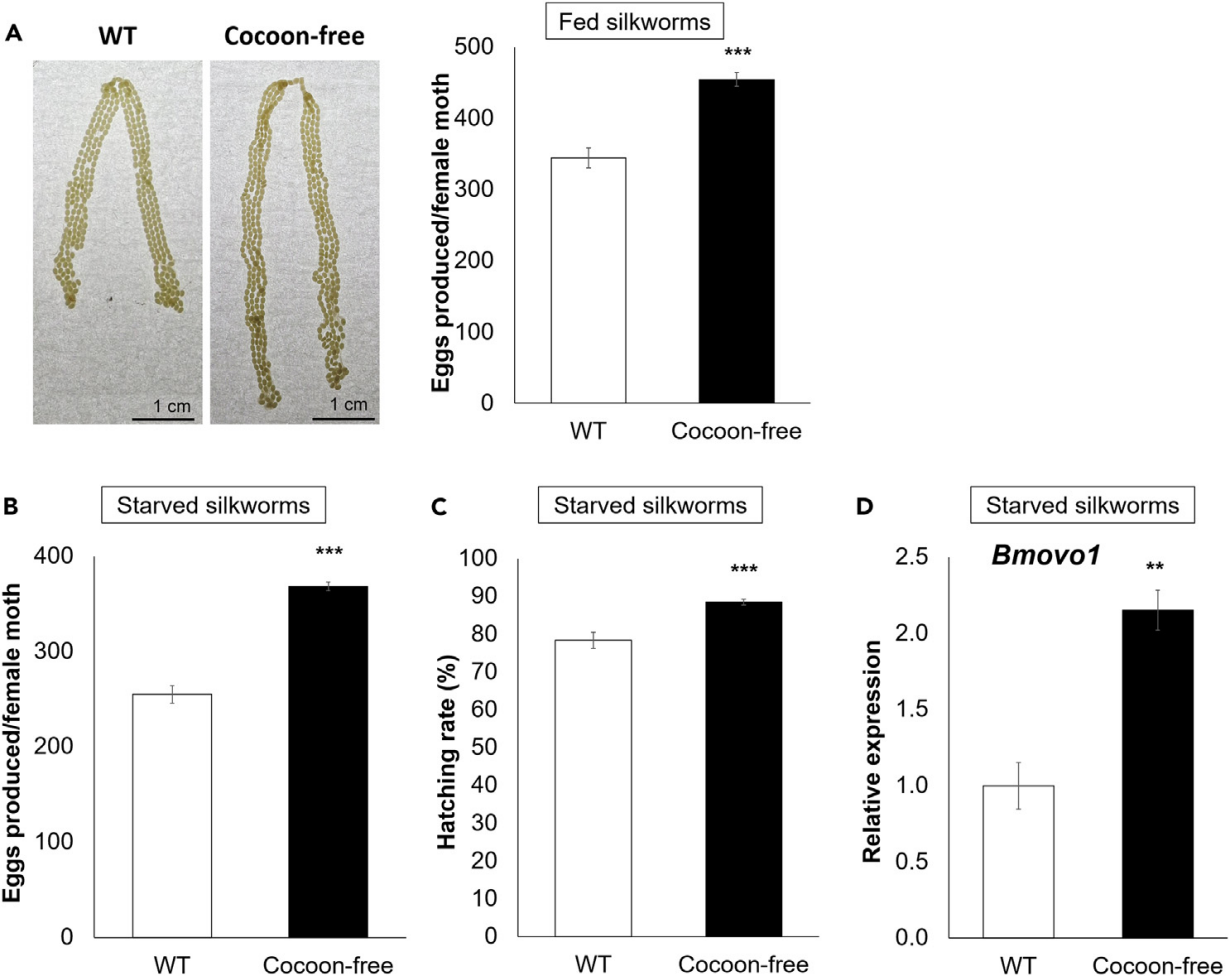
Enhanced energy reserves in the pupal fat body increased the reproductive success in the cocoon-free silkworms (A) Fecundity comparison between the adult female WT and cocoon-free silkworms whose larvae were fed *ad libitum* (n = 11). Representative images of the ovaries dissected from the female WT and cocoon-free silkworms immediately after eclosion are shown. (Scale bar, 1 cm). (B) Fecundity and (C) egg hatching rate comparison between the adult female WT and cocoon-free silkworms when their larvae were semi-starved (n = 20–24). (D) Expression levels of *Bmovo1* in the ovaries of the adult female WT and cocoon-free silkworms at day 0 post-pupation (pp) when the larvae were semi-starved. The mRNA levels were normalized to the *rp49* reference gene and expressed as fold-change relative to the WT (set as 1) (n = 3). Data represent mean ± SEM; ∗∗p < 0.01; ∗∗∗p < 0.001 according to the Student’s *t* test.

Examination of the ovaries at day 0 post-eclosion revealed fully developed ovaries in both the adult female WT and cocoon-free silkworms fed *ad libitum* as larvae, with the cocoon-free silkworms exhibiting longer ovaries (Figure 3A). Notably, the adult female cocoon-free silkworms who were fed *ad libitum* as larvae, displayed enhanced fecundity, producing a significantly higher number of eggs (455 ± 10) than the WT silkworms (345 ± 14) (p < 0.001; Figure 3A). Moreover, no differences in the egg hatching rate between the adult female WT (>99%) and cocoon-free (>99%) silkworms were observed when they were fed *ad libitum* as larvae. These findings indicate a relationship between reproductive output and nutrient availability in silkworms.

To further confirm the relationship between the resource availability and reproductive investment, we evaluated the fecundity and egg hatching rate between the adult female WT and cocoon-free silkworms when their larvae were reared under semi-starvation conditions. The data showed that fecundity of adult female WT and cocoon-free silkworms was reduced when the larvae were semi-starved (Figure 3B) compared to larvae that were fed *ad libitum* (Figure 3A). Notably, the decline in fecundity was more prominent in the adult female WT silkworms when their larvae were semi-starved (∼26%). In contrast, the adult female cocoon-free silkworms exhibited a milder reduction of ∼19% in fecundity when their larvae were semi-starved. Specifically, the adult female WT silkworms produced fewer eggs (255 ± 9) than those of the cocoon-free silkworms (368 ± 5) (p < 0.001; Figure 3B). The eggs laid by the adult female WT silkworms also exhibited a significantly lower hatching rate (∼78%) than those laid by the cocoon-free silkworms (∼89%) when they were semi-starved as larvae (p < 0.001; Figure 3C). These findings indicate that the cocoon-free silkworms, which accumulated more energy reserves during the larval stage because of the repression of silk protein synthesis, are more resistant to starvation stress compared to the WT silkworms.

The hatching rate and number of oviposited eggs are related to the *Bmovo1* gene^32^ thus, we determined whether the gene expression of *Bmovo*1 was influenced by starvation stress. The results obtained revealed that there were significantly lower levels of *Bmovo*1 mRNA (2.2-fold decrease, p < 0.01; Figure 3D) in the ovaries of female WT silkworm pupae compared to the female cocoon-free silkworm pupae at day 0 post-pupation (pp) when they were semi-starved as larvae. This finding indicates that the *Bmovo1* gene expression was influenced by starvation stress. The observed differences in the fecundity and egg hatching rate between the adult female WT and cocoon-free silkworms under the fed *ad libitum* and semi-starvation larval cultivation conditions emphasize the significance of resource availability and allocation in enhancing the reproductive success of silkworms.

### Increased vitellogenesis and oocyte growth promoted ovarian development

**Figure 4:**
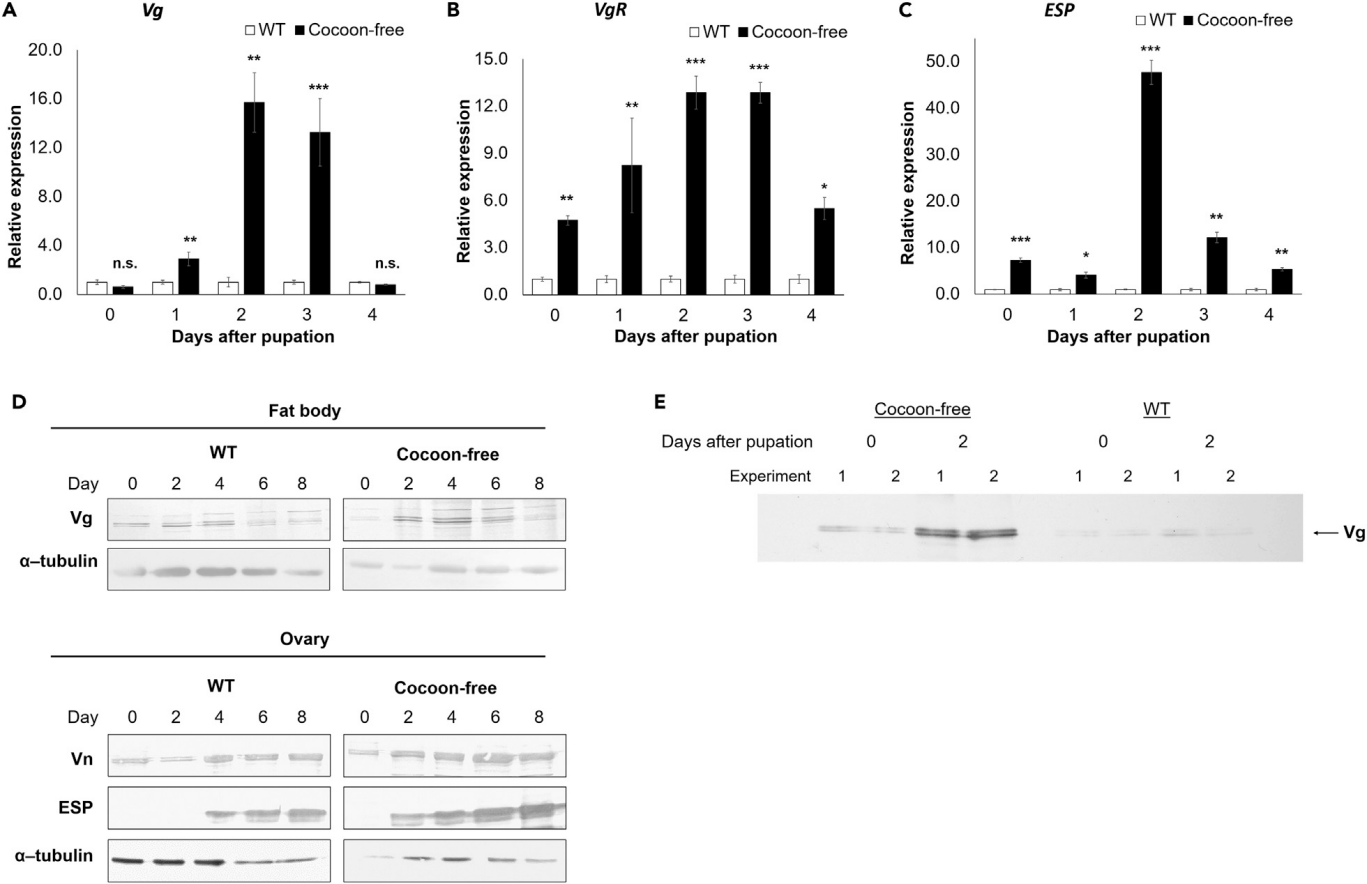
Increased vitellogenesis and oocyte growth promoted the ovarian development in cocoon-free silkworms Relative expression of the (A) *Vg* gene in the pupal fat bodies, and (B) *VgR* and (C) *ESP* genes in the ovaries of the female WT and cocoon-free silkworms at day 0–4 post-pupation (pp). The mRNA levels were normalized to the *rp49* reference gene and expressed as fold-change relative to the WT (set as 1) (n = 3). (D) Immunoblot analysis of the expression of egg yolk proteins in pupal fat bodies and ovaries of female WT and cocoon-free silkworms at different time points (day 0, 2, 4, 6, and 8 pp). The images were representative of three independent immunoblots, each involving biologically independent samples (n = 3). Figure S13 presents additional immunoblots. (E) Immunoblot analysis of Vg protein secreted during *in vitro* fat body culture from pupal fat bodies of female WT and cocoon-free silkworms using the specific anti-serum. Vg, vitellogenin; VgR, vitellogenin receptor; Vn, vitellin; ESP, egg-specific protein. Data represent mean ± SEM; n.s., not significant; ∗p < 0.05; ∗∗p < 0.01; ∗∗∗p < 0.001 according to the Student’s *t* test.

We analyzed the expression of the genes involved in vitellogenesis and oocyte growth by qRT-PCR. The ribosomal protein 49 (*rp49*), which showed low variation and high stability across diverse tissues during different pupal stages of WT and cocoon-free silkworms (Figure S12), was used as a reference gene for normalization (see STAR Methods for further information). In the female cocoon-free silkworm pupae, there was a general upregulation of the vitellogenin (*Vg*) mRNA in the fat bodies compared to the female WT silkworms, with a notable increase at day 1 and peaked at day 2 pp (Figure 4A). These data were supported by the immunoblot data from three independent experiments, which showed a similar trend of elevated Vg protein expression in the female cocoon-free silkworm pupal fat bodies from day 2 onwards and reaching its peak at day 4, before decreased by day 6 pp (Figures 4D and S13). The *in vitro* fat body culture experiments revealed a higher secretion of Vg protein into the medium by the female cocoon-free silkworm pupal fat bodies than that of the female WT silkworms (Figure 4E), which is subsequently taken up and stored as vitellin (Vn) in the developing oocytes.^33^ Immunoblot analysis revealed a trend of elevated Vn protein expression in the pupal ovaries of the female cocoon-free silkworms compared to that of the female WT silkworms (Figures 4D and S13). The Vg receptor (VgR) is essential for Vg uptake into the developing oocytes via endocytosis.^33^ Notably, as shown in Figure 4B, the *VgR* mRNA levels in the ovaries of the female cocoon-free silkworm pupae were significantly increased compared to those of female WT silkworms, with the highest detection level at day 2 and 3 pp. The egg-specific protein (ESP), a minor yolk protein compared to vitellin, is crucial for egg fertility.^34^ The *ESP* mRNA levels in the ovaries of the female cocoon-free silkworm pupae exhibited a similar trend as that of *Vg*, with peak transcription at day 2 compared to the female WT silkworms (Figure 4C). Specifically, ovarian development was accelerated in the female cocoon-free silkworm pupae, as the ESP was detected approximately 48 h earlier than in the female WT silkworms (Figures 4D and S13). In contrast, the ESP expression in the ovaries of female WT silkworm pupae was only observed after day 2, which is consistent with previous studies.^35^^,^^36^ Collectively, these data indicate that the increased egg yolk protein accumulation in the oocytes promotes reproductive efficiency in the female cocoon-free silkworm pupae.

### Insulin/insulin-like signaling (IIS) and target of rapamycin (TOR) signaling pathways were upregulated with increased nutrient availability

While ovarian development in insects such as *Aedes aegypti*^37^ and *Tribolium castaneum*^38^ is regulated by both juvenile hormone (JH) and 20-hydroxyecdysone (20E); in silkworms, this process is solely regulated by 20E.^39^^,^^40^ It is worth noting that the Broad-Complex isoform Z2 (BrCZ2), which plays a crucial role in the 20E-mediated transcriptional activation of the expression of *Vg*,^41^ showed a significant upregulation in the fat bodies of the day 0 female cocoon-free silkworm pupae compared to the female WT silkworms (3.1-fold, p < 0.01; Figure S14). Recent studies have revealed the involvement of the IIS and TOR signaling in the stimulation of the vitellogenesis in insects such as *Aedes* mosquitoes,^42^ aside from their role in regulating the growth and development in response to the insects nutritional status.^43^^,^^44^ However, their role in silkworm reproduction remains poorly understood. To identify a potential link between the IIS/TOR signaling pathways and reproduction in the cocoon-free silkworm, we assessed the mRNA levels of the IIS/TOR signaling pathway components in the pupal fat body using qRT-PCR analysis, with *rp49* that was most stably expressed across diverse tissues (Figure S12) as a reference gene for normalization.

**Figure 5:**
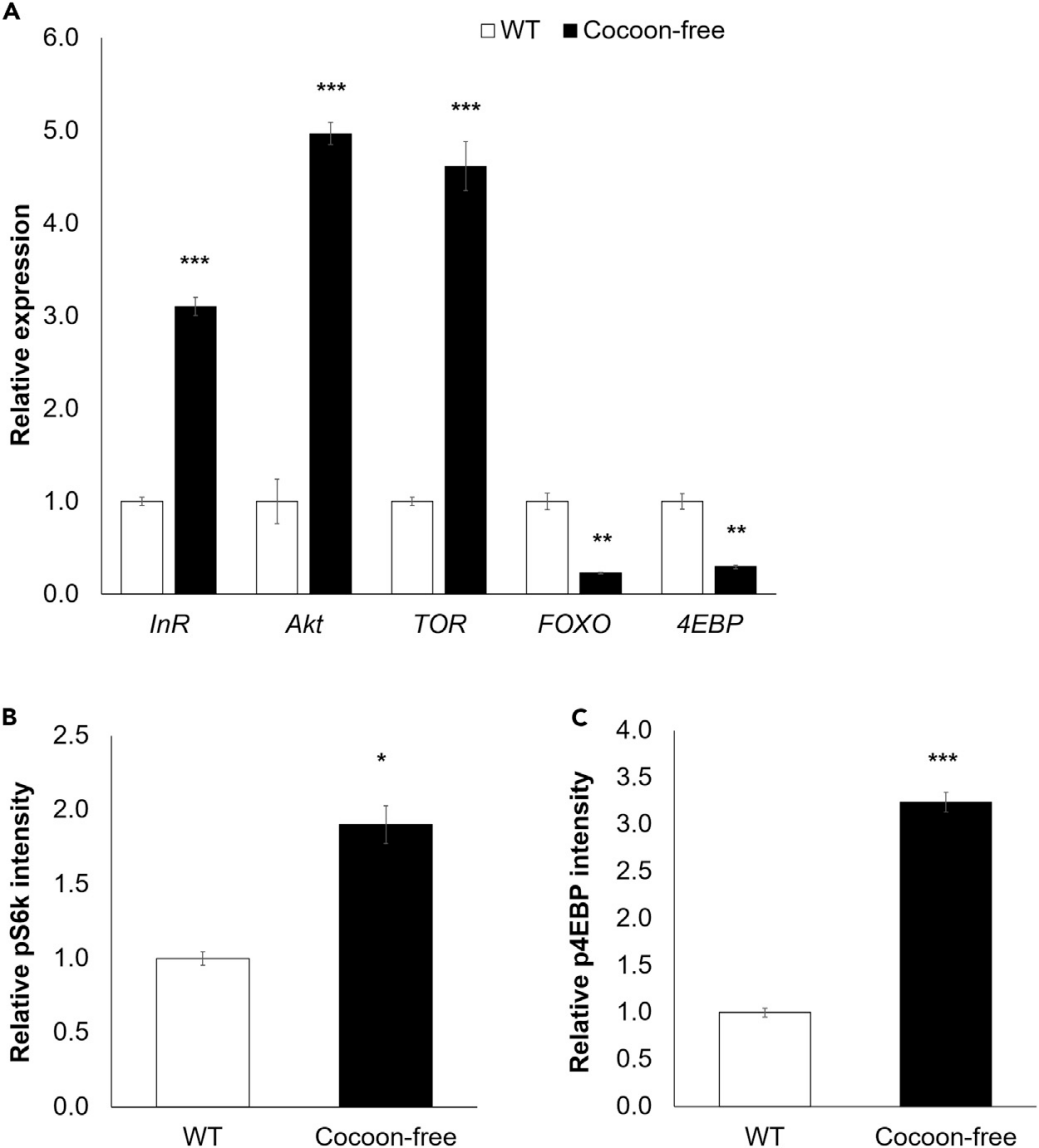
Increased energy reserves upregulated insulin/insulin-like signaling and target of rapamycin (IIS/TOR) signaling in the fat body of female cocoon-free silkworms and stimulated protein synthesis (A) The mRNA levels of IIS/TOR pathway components were normalized to the *rp49* reference gene and expressed as fold-change relative to the WT (set as 1) (n = 3). Expression levels of phosphorylated (B) S6K (pS6K) and (C) 4EBP (p4EBP) in the pupal fat bodies of female cocoon-free silkworms compared to the female WT silkworms (n = 3). The band intensity was normalized to α-tubulin and expressed as fold-change relative to the WT (set as 1). The immunoblot images are shown in Figure S16. Data represent mean ± SEM; ∗p < 0.05; ∗∗p < 0.01; ∗∗∗p < 0.001 according to the Student’s *t* test.

A significant upregulation of *InR* (insulin receptor), *Akt* (protein kinase B), and *TOR* gene expression was observed in the female cocoon-free silkworms. The mRNA levels of *InR*, *Akt*, and *TOR* in the pupal fat bodies of the female cocoon-free silkworms were 3.1-, 5.0-, and 4.6-fold higher, respectively, compared to the female WT silkworms (p < 0.001; Figure 5A). Conversely, the expression of the *FOXO* (Forkhead box protein O) and *4EBP* (4E-binding protein) genes were down-regulated in the female cocoon-free silkworms. The mRNA levels of *FOXO* and *4EBP* in the pupal fat bodies were decreased by 4.4- and 3.4-fold, respectively, in the female cocoon-free silkworms compared to the female WT silkworms (p < 0.01; Figure 5A). To further link elevated IIS/TOR signaling with enhanced reproduction processes such as vitellogenesis in cocoon-free silkworms, we treated cultured fat bodies of day 0 cocoon-free silkworm pupae with rapamycin. The findings showed an 11-fold reduction in the *Vg* mRNA levels in rapamycin-treated cocoon-free silkworm pupal fat bodies compared with the nontreated samples (p < 0.001; Figure S15).

A significant effect of TOR activation in response to nutritional stimuli is the regulation of protein synthesis through the phosphorylation of two effector molecules, S6K (S6-kinase) translational activator and 4EBP translational repressor. We evaluated the phosphorylation of S6K (pS6K) and 4EBP (p4EBP) in the pupal fat body using phospho-specific antibodies. Significantly higher levels of pS6K (1.9-fold, p < 0.05; Figures 5B and S16A) and p4EBP (3.0-fold, p < 0.001; Figures 5C and S16B) were detected in the pupal fat bodies of female cocoon-free silkworms compared with those of female WT silkworms. To further confirm that this increased protein synthesis in the cocoon-free silkworm pupae was a result of the nutrition-dependent activation of translational regulator mechanisms within the fat body, we injected a recombinant baculovirus expressing luciferase into the female WT and cocoon-free silkworm pupae. The fat body is known to be the primary site for baculovirus replication, particularly the nucleopolyhedrovirus.^45^ Using a luminescence assay, we measured the luciferase activity and demonstrated that the female cocoon-free silkworm pupae exhibited a 5.3-fold increase in luminescence than the female WT silkworms (p < 0.001; Figure S17). These results confirmed the enhanced protein translational capacity in the pupal fat body of the female cocoon-free silkworms, which could be further harnessed to increase the production yield of foreign proteins using recombinant baculovirus infection. Together, these findings suggest that IIS/TOR signaling is involved in the regulation of the ovarian development in the cocoon-free silkworm pupae via the augmentation of protein synthesis through the sensing of nutritional levels.

## Discussion

Resource allocation to the different traits in organisms can result in trade-offs when those traits depend on the same finite energy resource.^46^ Gaining insight into how silkworms manage and prioritize their limited energy resources can provide a mechanistic sense of resource allocation and elucidate the potential diversion of the energy resources for alternative purposes. In this study, we show that the abundance of amino acids and nutrient reserves in cocoon-free silkworms increases IIS/TOR signaling, leading to the upregulation of yolk protein synthesis at the transcriptional and translational levels, thereby promoting ovarian development and enhancing fecundity.

Interestingly, contrary to previous reports on inhibiting cocoon formation by surgical removal of silk glands or blocking spinnerets,^13^^,^^23^^,^^47^ no developmental defects in cocoon-free silkworms were detected, despite the accumulation of amino acids in larval hemolymph (Figure S10). The difference in the outcomes may be attributed to the accumulation of amino acids in cocoon-free silkworm larval hemolymph did not reach toxic levels that could cause negative effects.^23^^,^^25^ Moreover, Xin et al.^48^ demonstrated that the deletion of the transcription factor *Bmsage*, which is involved in silk gland development, resulted in malformed silk glands lacking MSGs and PSGs, which led to inhibited cocoon formation and pupation failure. Dissection of these deceased mutant silkworms revealed the presence of undigested leaves in the midgut, which indicates the disruption of nutrient processing and absorption in the absence of silk glands.^48^ Therefore, we speculate that there is a potential relationship between the silk glands and midgut in regulating nutrient balance and physiological processes in silkworms. However, this theory needs to be assessed in future research. Additionally, Takasu et al.^14^ reported that half of the first-generation TALEN-mediated *Ser1* knockdown pupae died before eclosion and the potential for further filial generations was not determined. The precise reason for this was unknown; however, a significant reduction (>2-fold) in the mean cocoon weight, resulting from the absence of sericin to facilitate fibroin exudation,^14^ suggests that silk protein retention may be harmful to silkworms. This notion is supported by a previous study, where the cauterization of the silkworm spinneret halted cocoon spinning but also resulted in silk protein retention and pupal lethality.^23^ In the case of cocoon-free silkworms, it is most likely that the minimal retention or absence of silk proteins, along with the presence of silk glands, prevented the pupation failure or pupal lethality.

Remarkably, our results demonstrated that cocoon-free silkworms underwent metamorphosis into pupae that were larger in size than those of the WT silkworms (Figure 2A). Although larval growth generally determines the final body size of insects,^49^ the minimal differences in the growth rate and developmental timing between WT and cocoon-free silkworms suggest the involvement of additional factors that influence the ultimate body size. Notably, we discovered a higher pupal dry weight (Figure S11C) and increased levels of glycogen and TAG in the fat body (Figures 2B and 2C) of cocoon-free silkworms. These observations indicate that reduced energy expenditure for silk production results in a shift in the mobilization of stored energy reserves. Our result is consistent with a prior study by Inagaki et al.,^29^ wherein the surgical removal of silk glands from silkworms reduced their metabolic rate, as inferred from the increased incorporation of radioactively labeled substrate into the fat body and reduced incorporation into the CO_2_ released during glucose oxidation. Therefore, it appears that the changes in silkworm body size are associated with increased accumulation of energy reserves.

Furthermore, nutritional status is strongly related to reproduction in *Aedes* mosquitoes and *Drosophila*. However, very little is known about silkworms. The increase in lipid stores in mosquitoes has been discovered to positively regulate yolk protein synthesis and oocyte development.^50^ Additionally, glycogen serves as an essential source of metabolic fuel for embryonic development.^51^ Moreover, earlier research reported that in situations of nutritional stress, egg development in mosquitoes and *Drosophila* can be halted or even resorbed.^52^^,^^53^ In this study, we have confirmed a strong correlation between the reproduction and energy reserves in silkworms, especially when they are nutritionally stressed. Greater availability of energy reserves improves resistance to starvation stress. Notably, the reduction in fecundity and egg hatchability due to nutritional deprivation was milder in cocoon-free silkworms compared with WT silkworms (Figures 3B and 3C). Given this, it is reasonable to propose that selective breeding for increased silk production in silkworms throughout the domestication process incurs a fecundity cost, albeit not to the extent of sterility. Expanding on this, it is conceivable that the development of protective cases such as cocoons in wild insects enhances parental survival, thereby reducing the offspring production, while maintaining an optimal balance of overall fitness.

The nutrient-sensing mechanisms play a crucial role in controlling vitellogenesis in insects, aside from hormonal signaling.^42^^,^^54^^,^^55^ In mosquitoes, egg development remains arrested until stimulated by nutrients acquired from blood feeding in the adult stage.^56^ In contrast, in silkworms, egg development is propelled by stored energy reserves acquired as larvae and retained after larval–pupal metamorphosis. Despite these differences, the nutritional status of insects generally determines their reproductive success. In mosquitoes, an increase in amino acids after a blood meal triggers TOR signaling, which in turn positively controls *Vg* expression.^55^ Additionally, the synergistic action of insulin and 20E signaling pathways has been found to be involved in stimulating *Vg* expression in mosquitoes.^54^ Interestingly, we discovered that the elements of the IIS/TOR signaling pathways were upregulated (Figure 5) and *Vg* expression was enhanced (Figure 4) in cocoon-free silkworms with higher hemolymph amino acid levels and nutrient reserves. The observation that the elevated *Vg* expression can be reversed by rapamycin treatment (Figure S15) further provides compelling evidence of a connection between amino acid-stimulated TOR upregulation and enhanced vitellogenesis in cocoon-free silkworms. However, a recent study showed that the mutation of insulin-like peptide (ILP) *ilp6* in mosquitoes impaired IIS signaling and resulted in aberrant ovarian development.^50^ While our study did not elucidate the activation of IIS by ILPs in the cocoon-free silkworms, a previous study identified an insulin-like growth factor-like protein, a homolog of *Drosophila* ILP, which is highly expressed in the silkworm pupal fat body and involved in regulating female genital organ development.^57^ Together, these data indicate that the cocoon-free silkworm pupal fat body detects increased nutritional levels, causing the release of ILPs, which activate the IIS pathway and, when combined with TOR pathway activation by amino acid signals, upregulate vitellogenesis.

Previously, it was elucidated that TOR stimulates *Vg* expression in mosquitoes through both transcriptional and translational regulatory mechanisms. Specifically, amino acid-mediated TOR activation or insulin stimulation increases the phosphorylation of S6K,^54^^,^^58^ leading to enhanced translation of the GATA transcription factor and, ultimately, increased transcription of *Vg*.^59^ A recent finding revealed the binding sites for GATA-type transcription factors in the proximal region of *Vg* gene in silkworms.^60^ Although the expression of GATA was not specifically examined in this study, the increased levels of pS6K in pupal fat bodies (Figure 5B) and elevated *Vg* transcript levels (Figure 4A) in cocoon-free silkworms suggest a potential relationship between GATA and IIS/TOR signaling in the regulation of silkworm vitellogenesis. Furthermore, 4EBP, another key downstream effector molecule of TOR with a major function in modulating translational events similar to S6K, may also play a crucial role in regulating vitellogenesis. It is worth noting that TOR activation necessary to promote translation is associated with increased phosphorylation of 4EBP and a reduction in transcript levels.^61^ This pattern aligns with our observation of high p4EBP (Figure 5C) and low 4EBP mRNA levels (Figure 5A) in cocoon-free silkworms. Thus, it appears that increasing 4EBP phosphorylation encourages its repression, and in combination with increased pS6K, it leads to an upregulation of the translation rate in cocoon-free silkworms. Additionally, the increased expression of Br transcription factor, which promotes *Vg* transcription in response to 20E stimulation,^41^ in cocoon-free silkworms suggests a dual regulatory mechanism involving both hormonal and nutritional signaling in the regulation of vitellogenesis in silkworms.

Another intriguing aspect of cocoon formation inhibition is the elimination of the desilking step required for pupae to be released from their cocoons. This trait holds significance with the increasing interest in the use of silkworms as living biofactories to manufacture recombinant therapeutic or viral proteins,^62^ as well as their potential use as livestock feed because of their high protein and fat content.^63^ We have demonstrated high expression levels of the recombinant luciferase protein via baculovirus infection using cocoon-free silkworms (Figure S17). With pupae, scalable production is also made significantly easier, and manual labor is reduced because of their non-mobility, soft body cuticle, and automation of pupae handling and virus inoculation.^64^ Furthermore, the expression of P1A269 in cocoon-free silkworms raises minimal safety concerns regarding their use as a biofactory. This is explained by the absence of a secretory signal sequence preventing release from silk glands and a lack of a putative receptor-binding domain that prevents the binding and internalization of P1A269 into cells.^17^ Consequently, cocoon-free silkworms present a promising and safe host for biomanufacturing.

Evidence of enhanced energy allocation to egg development through repression of silk protein synthesis and interference with energy storage mobilization offers important insights into the dynamic nature of energy control in other organisms. Cocoon-free silkworms serve as a model organism, exemplifying how enhancement of certain organ functions comes at the cost of repressing others, highlighting the potential for trade-offs in organ function within biological systems. Such knowledge is pivotal for developing effective breeding techniques in agriculture by reallocating energy resources from nonessential tasks to emphasize economically important features, thereby maximizing productivity. Moreover, the enhanced fecundity of cocoon-free silkworms enables the generation of a substantial quantity of larvae or pupae as biofactories, thereby facilitating efficient and scalable mass production of recombinant proteins. The increased protein synthesis capacity resulting from the upregulation of IIS/TOR signaling in the cocoon-free silkworm pupal fat bodies presents the possibility of using this machinery to further boost the recombinant protein yield. The potential applications described above enhance the value of silkworms, allowing them to surpass their traditional agricultural significance.

### Limitations of the study

While TOR signaling pathway is upregulated in the cocoon-free silkworms, whether that was induced by specific amino acid constituents of silk proteins accumulated in the hemolymph is still unclear and will require further validation. Whether the IIS pathway is activated by insulin or insulin-like growth factors secreted in response to elevated glycogen levels in the fat body of cocoon-free silkworms is also not clarified and awaits further investigation. Our study elucidated that the higher fecundity of cocoon-free silkworms is due to increased protein synthesis capacity in the pupal fat body caused by the IIS/TOR signaling upregulation, but potential crosstalk with ecdysone signaling was not evaluated. Further studies are required to gain a comprehensive understanding of this dual regulatory mechanism in silkworm reproduction regulation.

## STAR★Methods

### Key resources table

**Table:** 

REAGENT or RESOURCE	SOURCE	IDENTIFIER
**Antibodies**		

Goat anti-rabbit IgG (H+L), HRP conjugated	BioRad	Cat#1706515; RRID:AB_11125142
Goat anti-mouse IgG (H+L), HRP conjugated	BioRad	Cat#1706516; RRID: AB_2921252
Anti-FLAG (DYKDDDK), HRP conjugated	Sigma-Aldrich	Cat#A8592; RRID:AB_439702
Rabbit anti-phospho-p70 S6 Kinase	Cell Signalling	Cat#9205; RRID: AB_330944
Rabbit anti-phospho-4E-BP1 (Thr37/42)	Cell Signalling	Cat#2855; RRID:AB_560835
Rabbit anti-α-Tubulin	Proteintech	Cat#66031-1-Ig; RRID: AB_11042766
Rabbit anti-Vg	A gift from Dr. Kunihiro Shiomi and Dr. Toshinobu Yaginuma	N/A
Rabbit anti-ESP		N/A

**Bacterial and virus strains**		

BmNPV-luciferase virus	Nihon Nosan Co. Ltd.	N/A

**Chemicals, peptides, and recombinant proteins**		

Grace’s medium	Thermo Fisher Scientific	Cat#11300027
Fetal bovine serum	MP Biomedicals	Cat#092910154
Sau3AI	NEB	Cat#R0169S
T4 DNA Ligase	Toyobo	Cat#LGA-111
Lithium bromide	FUJIFILM Wako Pure Chemical	Cat#129-01125
EZApply SDS-PAGE sample buffer	ATTO Co.	Cat#2332330
e-PAGEL 5%–20% gradient gel	ATTO Co.	Cat#2331830
Ethanol	Nacalai Tesque	Cat#14712-63
Sodium chloride (NaCl)	FUJIFILM Wako Pure Chemical	Cat#191-01665
Chloroform	Nacalai Tesque	Cat#08426-13
Methanol	Nacalai Tesque	Cat#21914-03
Acetic acid	Nacalai Tesque	Cat#00212-43
Triton X-100	Nacalai Tesque	Cat#35501-02
Glycerol Standard Solution	Sigma-Adrich	Cat#G7793
ISOGEN II	Nippon Gene	Cat#311-07361
Protease inhibitor cocktail	Nacalai Tesque	Cat#03969-34
RIPA buffer	Nacalai Tesque	Cat#16488-34
Phosphatase inhibitor cocktail, EDTA free	Nacalai Tesque	Cat#07575-51
Blocking One	Nacalai Tesque	Cat#03953-95
Blocking One-P	Nacalai Tesque	Cat#05999-84
Chemi-Lumi One L	Nacalai Tesque	Cat#07880-54
Rapamycin	Calbiochem	Cat#553211

**Critical commercial assays**		

In-fusion HD Cloning Kit	Takara Bio	Cat#639649
Plasmid Midiprep Kit	Qiagen	Cat#12143
BCA protein assay kit	Thermo Fisher Scientific	Cat#23225
Glycogen Assay Kits	Cell Biolabs	Cat#MET-5022
Serum Triglyceride Determination Kit	Sigma-Aldrich	Cat#TR0100
SuperScript III Platinum SYBR Green One-Step qRT-PCR kit	Invitrogen	Cat#11736059
Peroxidase Stain DAB Kit (Brown Stain)	Nacalai Tesque	Cat#25985-50
PicaGene BrillianStar-LT luminescence kit	Toyo Ink	Cat#306-04331
DNA Extractor® TIS Kit	FUJIFILM Wako Pure Chemical	Cat#296-67701

**Experimental models: Cell lines**		

Bombyx mori-derived BmN cells	N/A	N/A

**Experimental models: Organisms/strains**		

Silkworm: non-diapausing wildtype silkworm line *w1-pnd* line	N/A	N/A
Silkworm: w1-pnd^P1A269/P1A269^ line	Otsuki et al.^18^	N/A

**Oligonucleotides**		

See Table S1		

**Recombinant DNA**		

pIZ-Ser1Pro-H1/P1A269/FLAG	This study	N/A
pBacMCS[Ser1Pro-P1A269, 3 × P3-DsRed]	This study	N/A

**Software and algorithms**		

ImageJ	NIH	https://imagej.nih.gov/ij/; RRID:SCR_003070
SigmaPlot 12.0	Systat Software	https://systatsoftware.com/sigmaplot/; RRID:SCR_003210

### Resource availability

#### Lead contact

Further information and requests for resources and reagents should be directed to and will be fulfilled by the lead contact, Eiji Kotani (kotani@kit.ac.jp)

#### Materials availability

This study did not generate new unique reagents. All key resources are listed in the key resources table.

#### Data and code availability

• This paper does not report original code.
• All data are included in the article and/or supplemental information.
• Any additional information required to reanalyze the data reported in this paper is available from the lead contact upon request.

### Experimental model and subject details

#### Insects

The non-diapausing wildtype silkworm line *w1-pnd* (WT) and a previously established *w1-pnd*^P1A269/P1A269^ line expressing the EGFP fluorescent marker (referred to as the fibroin-free cocoon line in this work),^18^ were used in this study. All silkworm larvae were reared on an artificial diet (120 g) from Nihon Nosan Co., Ltd. and maintained at 27°C from the first to third instars. From the fourth instar onwards, unless otherwise specified, the silkworm larvae were reared at a density of 60 per 135 cm^2^ case and fed *ad libitum* (artificial diet (120 g) from Kimono Brain Co., Ltd.; replenished every 2 days). The tissue samples used in this study were extracted from randomly selected silkworms that were fed *ad libitum* as larvae unless otherwise stated. For the semi-starvation study, the silkworm larvae were reared at a density of 120 per 135 cm^2^ case from the fourth instar onwards, with the artificial diet (120 g) that was replenished every 5 days.

#### Insect cell line

*B. mori*-derived BmN cells were cultured in Grace’s medium (Thermo Fisher Scientific) supplemented with 10% fetal bovine serum (FBS; MP Biomedicals) at 27°C and were subsequently used for virus infection and virus titer determination by the limiting dilution method.

### Method details

#### Donor plasmid construction

The oligonucleotide primer sequences used for plasmid construction are listed in Table S1. A DNA fragment encoding for the promoter and 5ʹ-untranslated region [from −579 to +59 bp] of the silkworm *Ser1* gene (accession no. DQ354392) was PCR amplified from WT genomic DNA with the primers Ser1fKpnI and Ser1rBamHI. The resulting PCR fragment was double-digested with *Kpn*I and *BamH*I enzymes, gel-purified, and cloned into the corresponding sites upstream of the P1A269 (N-terminal 269 amino acid sequence of pierisin-1A) fusion sequence in the pIZ-H1/P1A269/FLAG plasmid as previously reported,^18^ to produce the pIZ-Ser1Pro-H1/P1A269/FLAG plasmid (Figure S1A). The P1A269 fusion sequence comprises of an N-terminal cypovirus polyhedrin H1 with C-terminal FLAG (DYKDDDK) tags to facilitate detection with antibodies.^18^

For the construction of the donor plasmid pBacMCS[Ser1Pro-P1A269, 3 × P3-DsRed] (Figure S1B), the fusion sequence containing the P1A269, *Ser1* promoter, and OpIE2 polyadenylation signal was PCR amplified using the Ser1-P1Af and Ser1-P1Ar primers. This fragment was subcloned into the *Bg1*II-*EcoR*I sites of the pBacMCS[UAS, 3 × P3-egfp],^65^ along with the synthesized 3 × P3-DsRed fragment (accession number: AB713995), using the In-fusion HD Cloning Kit (Takara Bio). The resulting donor plasmid was purified using a Plasmid Midiprep Kit (Qiagen). All constructed plasmids were verified through DNA sequencing.

#### Generation of cocoon-free silkworm line

Silkworm germline transgenesis was performed by microinjection as previously described.^66^^,^^67^ The constructed pBacMCS[Ser1Pro-P1A269, 3 × P3-DsRed] (Figure S1B) donor plasmid and helper plasmid expressing the transposase were co-injected into the pre-blastoderm embryos of the WT line at 2–6 h after oviposition. The resulting G0 adults were sibling mated, and their G1 progeny were examined for the presence of DsRed-fluorescing eyes and nerve cord during the late embryonic and early fourth instar larval stages using a stereoscopic epifluorescence microscope (SZX16; Olympus). The G1 individuals that exhibited the desired red-eye phenotype were individually crossed with virgin WT adults to establish stable heterozygous transgenic lines, which maintained the transgene in their genetic background similar to that of the WT line. The individuals from the heterozygous transgenic lines were mated with each other for several generations to obtain a putative homozygous transgenic line, based on the intensity of red-eye fluorescence. The same strategy was utilized to establish the homozygous fibroin-free cocoon line.

The transgene genomic insertion site was validated by inverse PCR, following the previously described methods (see Table S2),^66^^,^^67^ which confirmed their homozygosity. Genomic DNA was isolated from the silk glands of the G2 fifth instar larvae of the Ser1-free cocoon line using standard phenol-chloroform extraction, after overnight incubation with proteinase K at 37°C, and an additional incubation with RNase for 3 h at 25°C. The extracted genomic DNA was digested with *Sau*3AI (NEB) and circularized by overnight incubation with T4 DNA Ligase (Toyobo) at 16°C. Subsequently, the DNA fragments located in the vicinity of both the up- and downstream regions of the *piggyBac* inverted terminal repeats were PCR-amplified using primers (see Table S1 for primer sequences) and subjected to sequencing for confirmation. The resulting sequences were analyzed using BLAST searches against the silkworm genome database via KAIKOBLAST (https://sgp.dna.affrc.go.jp/KAIKObase/).

To generate the cocoon-free silkworm line, the Ser1-free cocoon line was crossed with the fibroin-free cocoon line. The resulting progeny from this crossbreeding were screened for the presence of DsRed- and EGFP-fluorescing eyes and nerve cord at the late embryonic and early fourth instar larval stages. Individuals exhibiting both DsRed and EGFP-fluorescing eyes were selected and bred to homozygosity to establish the cocoon-free silkworm line.

#### SDS-PAGE analysis of silk proteins in cocoons

Protein contents of the transgenic silkworm cocoons were analyzed by SDS-PAGE as previously described,^68^ with slight modifications. One hundred milligrams of cocoons from the Ser1-free cocoon silkworm line were dissolved in 2 mL of 8 M lithium bromide (FUJIFILM Wako Pure Chemical Industries, Ltd.) at 37°C for 3 h, followed by overnight incubation at 4°C. The resulting silk solutions were diluted 33-fold in pure water, combined with an equal volume of EZApply SDS-PAGE sample buffer (ATTO Co.), and heated for 5 min at 95°C. Similar procedures were performed for the cocoons from the WT and fibroin-free cocoon lines. Equal amounts of protein (15 ng) from each sample were resolved on a 5%–20% gradient gel (e-PAGEL; ATTO Co.) and visualized with Coomassie Brilliant Blue staining.

#### Amino acid composition of larval hemolymph

Hemolymph (100 μL) was collected from the WT and cocoon-free silkworm larvae 2 days after the gut purge by cutting the abdominal legs. The collected hemolymph was immediately mixed with 3 volumes of chilled absolute ethanol (Nacalai Tesque) and centrifuged at 17,800 ×*g* for 20 min at 4°C. The amino acid composition of the ethanol-precipitated hemolymph samples was analyzed using Prominence HPLC (Shimadzu).

#### Morphology analysis and physiological characterization

Silk glands of day 6 fifth instar WT and cocoon-free silkworm larvae were dissected in 0.85% saline, dried, and weighed. Morphological analysis of the silk glands was performed using a stereoscopic microscope, and images were captured. For PCD analysis, the nuclei in different sections of silk glands dissected from day 7 fifth instar larvae and day 0 pupae of WT and cocoon-free silkworms were stained with DAPI as previously described,^21^ and visualized using a microscope (20× objective lens) equipped with fluorescence system (IX73; Olympus). To detect DNA fragmentation, DNA extracted from the respective sections of silk glands from WT and cocoon-free silkworms on the indicated days, using the DNA Extractor® TIS Kit (FUJIFILM Wako Pure Chemical), was electrophoresed on 2% agarose gels and visualized. Weight loss during the larval-pupal metamorphosis was compared by weighing the gut purge stage larvae and day 0 pupae of the male and female WT and cocoon-free silkworms, which were anesthetized with ice. Body size variations between the male and female WT and cocoon-free silkworms were assessed by capturing digital images using a camera (MX-1; Pentax). The water content and dry weight of the WT and cocoon-free silkworm pupae were compared after lyophilization by freeze drying. The cocoon-spinning behaviour of the WT and cocoon-free silkworms during the larval-pupal metamorphosis was investigated using a time-lapse video recording (TLC 200 Pro; Brinno Inc.). Fecundity was determined by counting the number of eggs produced by adult female WT and cocoon-free silkworms, considering both the total number of laid eggs and those remaining in the ovary of the moth. The fecundity was compared between the adult WT and cocoon-free silkworm females that were fed or semi-starved during larval stages. To further investigate the effect of the nutrient deprivation on the adult WT and cocoon-free silkworm females, the egg hatching rate and expression level of the *Bmovo1* gene in the ovary at day 0 pp were measured.

#### Metabolic characterization

Fat bodies (20–40 mg) were dissected from the pupae of the female WT and cocoon-free silkworms that were fed *ad libitum* as larvae at day 0 pp, rinsed twice with 0.85% saline, and stored at −80°C until analysis.

For the glycogen measurement, the fat bodies were homogenized in 120 μL of 1× PBS and were immediately heat-inactivated for 10 min at 70°C. Samples were centrifuged at 15,800 ×*g* for 3 min at 4°C. The resulting supernatants (20 μL) were used for the determination of the total soluble protein concentration using a BCA protein assay kit (Thermo Fisher Scientific) according to the manufacturers’ instructions. The remaining supernatant (100 μL) was diluted 1:30 with 1× PBS and used for glycogen measurement with a colorimetric-based glycogen assay kit (Cell Biolabs) following the manufacturer’s instructions. Briefly, quadruplicate 50 μL aliquots of each diluted sample were loaded into a 96-well plate along with duplicate glycogen standards that were provided in the kit. Furthermore, 10 μL of amyloglucosidase was added to the serially-diluted glycogen standards and half of the samples, while 1× PBS was added to the other half of the samples. The plate was incubated for 30 min at 37°C. A reaction mix (50 μL) containing horseradish peroxidase (HRP), glucose oxidase, and colorimetric probe was added to the samples, and the plate was incubated in the dark for a further 45 min at 37°C. The absorbance was measured at 540 nm using an iMark microplate reader (BioRad) and the sample glycogen concentrations were determined by subtracting the values of the PBS-treated samples from the values of the amyloglucosidase-treated samples.

For TAG measurement, the fat bodies were prepared following the same procedure as described for the glycogen measurement, except with the addition of the lipid extraction step using chloroform/methanol (1:2, v/v; Nacalai Tesque) before heat inactivation. Aliquots (20 μL) of the fat body homogenates were used for the soluble protein quantification with a BCA assay. The remaining homogenates (100 μL) underwent lipid extraction according to the method described by Bligh and Dyer^69^ with some modifications. Briefly, sequential addition of 375 μL chloroform/methanol (1:2, v/v), 125 μL chloroform, 0.5 M NaCl, and 0.5% (v/v) acetic acid (Nacalai Tesque) was performed, with vigorous vortexing after each addition, followed by centrifugation at 600 ×*g* for 10 min at 4°C. The lower phase containing the lipids was transferred to new tubes, dried under reduced pressure, and resuspended in 0.2% Triton X-100 (Nacalai Tesque) to the desired volume. The resuspended lipids were heat-inactivated for 10 min at 70°C before TAG quantification using the Serum Triglyceride Determination Kit (Sigma-Aldrich). A triolein equivalent glycerol standard (Sigma-Aldrich) was used as the standard. Quadruplicate aliquots (20 μL) of each sample and duplicate aliquots of the triolein standards were added to the microcentrifuge tubes. Two of the sample tubes received 20 μL of 0.2% Triton X-100, while the remaining sample tubes and triolein standards received the triglyceride reagent. All tubes were incubated for 5 min at 37°C, after which the absorbance was measured at 540 nm using an iMark microplate reader (BioRad). The TAG concentrations in the samples were determined by subtracting the values of the Triton X-100-treated samples from the values of the triglyceride reagent-treated samples. All measurements were normalized to the total soluble protein concentration as determined using the BCA assay kit. The data were analyzed using the Student’s *t*-test.

#### Quantitative RT-PCR (qRT-PCR)

Total RNA was extracted from the various silkworm tissues at different developmental stages using ISOGEN II (Nippon Gene). Specifically, the silk glands from day 2, 4, and 6 fifth instar larvae, as well as fat bodies and ovaries from day 0, 1, 2, 3, and 4 pupae were collected from both female WT and cocoon-free silkworms that were fed *ad libitum* as larvae. The qRT-PCR reactions were performed using the SuperScript III Platinum SYBR Green One-Step qRT-PCR kit (Invitrogen) as per the manufacturer’s instructions, in a CFX96™ real-time detection system (BioRad). Cycling parameters used were 1× 50°C for 3 min 30 s, 1× 95°C for 5 min, and 40× 95°C for 15 s and 60°C for 30 s with SYBR measurement, and a melting curve from 65°C–95°C increasing by 0.5°C every 5 s. Each qRT-PCR reaction was performed in a 10 μL reaction volume with 0.1 ng/μL RNA, using gene-specific primers (see Table S1 for primer sequences). All reactions were run in triplicate using three biologically independent samples. The mRNA relative fold-change was determined using the 2^−ΔΔCt^ method.^70^

For the gene expression analysis in the silk glands, the expression of the sericin genes (*Ser1*, *Ser2*, and *Ser3*) and fibroin genes (*FibH* and *FibL*) were normalized to the reference gene *18S rRNA* (Figure S5). In the case of the gene expression analysis of the reproduction-related genes (*Vg*, *VgR*, *ESP*, and *Bmovo1*), the *B. mori rp49* was selected for use as the reference gene. This selection was made based on the demonstration of the lowest stability measure (M value), as well as the coefficient of variation (CV) and standard deviation (SD) of Ct values amongst the candidate reference genes across diverse tissues during different pupal stages (Figure S12), as determined using the geNorm^71^ and BestKeeper^72^ programs, respectively. Similarly, for the gene expression analysis of the genes related to nutritional and hormonal signaling (*InR*, *Akt*, *TOR*, *FOXO*, *4EBP,* and *BrCZ2*), the *rp49* was used as the reference gene.

#### *In vitro* fat body culture

Fat bodies were dissected from the female WT and cocoon-free silkworm pupae at day 0 and 2 pp in cold, autoclaved 0.85% saline, and thoroughly rinsed. The pupal fat bodies (approximately 100 mg in total from three independent individuals) were incubated in 500 μL of Grace’s medium without FBS. The experiments were performed in duplicate. Following incubation for 60 min at 27°C, the culture medium was collected, centrifuged, and subjected to chloroform-methanol precipitation. Each precipitated sample was resuspended in 1× PBS containing a protease inhibitor cocktail (Nacalai Tesque) and the total protein concentration was determined using a BCA assay kit prior to performing immunoblot analysis. For rapamycin treatment, fat bodies (50 mg) dissected from three independent individuals of day 0 female cocoon-free silkworm pupae were cultured in either FBS-free Grace’s medium with or without 150 nM rapamycin (diluted from 200μM dissolved in DMSO) (Calbiochem) for 3 h at 27°C. Collected fat bodies were then subjected to RNA extraction using ISOGEN II and qRT-PCR analysis as described above.

#### Immunoblot

For the analysis of Vg, Vn, and ESP, the fat bodies and ovaries of day 0, 2, 4, 6, and 8 female WT and cocoon-free silkworm pupae were extracted and homogenized in 1× RIPA buffer (Nacalai Tesque) containing only a protease inhibitor cocktail. For the analysis of p4EBP and pS6K, the fat bodies of day 0 female WT and cocoon-free silkworm pupae were extracted and homogenized in 1× RIPA buffer containing protease and phosphatase inhibitor cocktails (Nacalai Tesque). For the analysis of P1A269, the MSGs dissected from day 6 fifth instar WT and cocoon-free silkworm larvae were homogenized in 1× RIPA buffer containing only a protease inhibitor cocktail. After homogenization, all samples were incubated for 30 min on ice and centrifuged at 4,400 ×*g* for 30 min at 4°C. The resulting supernatant was collected, and the total soluble protein content was determined using the BCA assay. Equal amounts of protein (100 μg for the p4EBP and pS6K and 3 μg for the egg yolk protein detection) from each sample were separated on a 12.5% SDS-PAGE, transferred to polyvinylidene fluoride (PVDF) membranes, and blocked for 1 h with Blocking One or Blocking One-P solution (Nacalai Tesque). The membranes were then incubated with their respective primary antibodies diluted with 1× PBS containing 0.05% Tween20.

For p4EBP and pS6K detection, overnight incubation with p4EBP antibody and pS6K antibody, respectively, (1:1000) (Cell Signaling) at 4°C was performed followed by incubation with HRP-conjugated goat-anti-rabbit (H+L) IgG secondary antibody (1:1000) (BioRad) for 3 h at 25°C. For Vg and ESP detection, 1 h incubation with anti-sera against Vg and ESP (1:25000) (kindly provided by Dr. Kunihiro Shiomi at Shinshu University and Dr. Toshinobu Yaginuma at Nagoya University, respectively) at 25°C was performed, followed by incubation with HRP-conjugated goat-anti-rabbit (H+L) IgG secondary antibody (1:5000) for 1 h at 25°C. For P1A269 detection, overnight incubation at 4°C with HRP-conjugated anti-FLAG (DYKDDDK) tagged antibody (1:5000) (Sigma-Aldrich) was performed. Tubulin protein used as loading control was detected with α-tubulin antibody (1:10000) (Proteintech) followed by an anti-mouse secondary antibody.

The immunoreactive bands were visualized with DAB substrate or Chemi-Lumi One L (Nacalai Tesque). The optical density of the protein bands was quantitated using computer-assisted analysis software, ImageJ (NIH; https://imagej.nih.gov/ij/), and the values were normalized to α-tubulin.

#### Luciferase reporter assay

BmNPV-luciferase virus, which carries the luciferase gene under the control of the polyhedrin promoter, was obtained from Nihon Nosan Co. Ltd. The virus was amplified in BmN cells, and the titer was determined using the limiting dilution method. A group of day 0 WT or cocoon-free silkworm pupae (*n* = 5) was injected with 50 μL of BmNPV-luciferase at a concentration of 2 × 10^13^ pfu/mL. Five days after the injection, each of the infected silkworm pupa was homogenized in 5 mL of 1× PBS and centrifuged at 4°C for 30 min at 4,400 ×*g* to remove the large debris. The resulting supernatant was assayed for luciferase activity using the PicaGene BrillianStar-LT luminescence kit (Toyo Ink). An aliquot of 50 μL from the 5000× diluted supernatant was mixed with a 50 μL of luciferin substrate reagent and incubated at 25°C for 5 min. The luminescence was measured using the GloMax Navigator System G2000 (Promega), and the relative luminescence unit value for the female cocoon-free silkworm pupae was expressed as a percentage of the female WT silkworm pupae.

### Quantification and statistical analysis

All statistical analyses were performed with SigmaPlot 12.0 (Systat Software, CA, USA). Data are represented as mean ± standard error of the mean (SEM). The number of biological replicates in experiments is indicated in the figure legends. All statistical analyses were compared with WT control groups and the significance was analyzed using the Student’s *t*-test at the following significance levels: ∗ *p* < 0.05; ∗∗ *p* < 0.01; ∗∗∗ *p* < 0.001.
