# Supplementary material for: Cytotoxin-mediated silk gland organ dysfunction diverts resources to enhance silkworm fecundity by potentiating nutrient-sensing IIS/TOR pathways
Source: iScience. 2024 Jan 11;27(2):108853. doi: 10.1016/j.isci.2024.108853 (PMC10830876; doi:10.1016/j.isci.2024.108853)
Supplement: Document S1. Figures S1–S17 and Tables S1 and S2 [file mmc1.pdf]

iScience, Volume *27*

Supplemental information

Cytotoxin-mediated silk gland organ dysfunction diverts resources to enhance silkworm fecundity by potentiating nutrient-sensing IIS/TOR pathways

Ping Ying Lye, Chika Shiraki, Yuta Fukushima, Keiko Takaki, Mervyn Wing On Liew, Masafumi Yamamoto, Keiji Wakabayashi, Hajime Mori, and Eiji Kotani

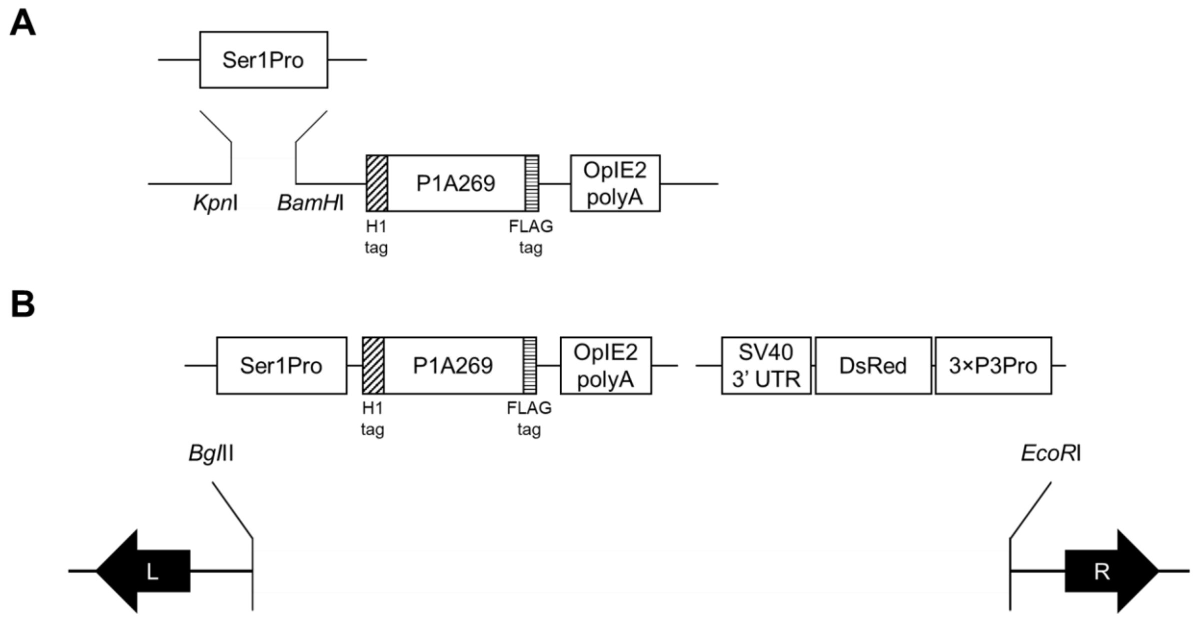

Figure S1. Schematic representation of the vector constructs used in the silkworm transgenesis. Related to STAR Methods.
(A) pIZ-Ser1Pro-H1/P1A269/FLAG was constructed by cloning into the *Kpn*I and *BamH*I-digested pIZ-H1/P1A269/FLAG plasmid, a 632 bp DNA fragment containing the *Ser1* promoter and 5'-untranslated region.
(B) pBacMCS[Ser1Pro-P1A269, 3 × P3-DsRed] was constructed by cloning into the *Bg1*II and *EcoR*1-digested pBacMCS[UAS, 3 × P3-egfp] plasmid, the DNA fragment containing the P1A269 fusion sequence with *Ser1* promoter, and OpIE2 polyadenylation signal from pIZ-Ser1Pro-H1/P1A269/FLAG along with the synthesized 3 × P3-DsRed fragment. The L and R black arrows refer to the *piggyBac* left and right inverted terminal repeats, respectively.

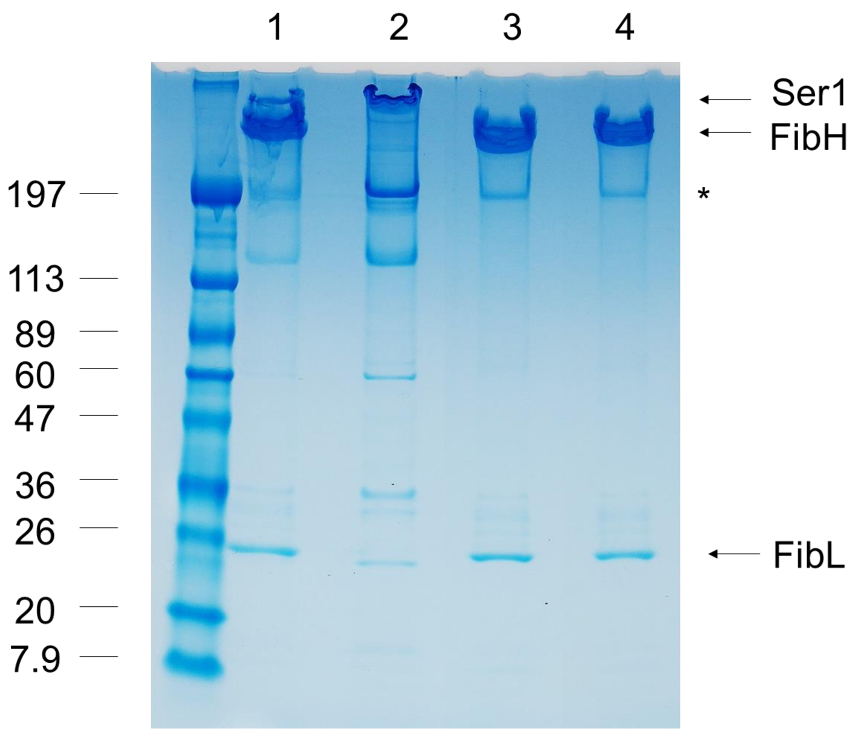

Figure S2. Characterization of the silk proteins present in the cocoons produced by WT, fibroin-free cocoon, and Ser1-free cocoon silkworms. Related to STAR Methods.
Silk proteins extracted from the cocoons of WT (lane 1), fibroin-free cocoon (lane 2), and Ser1-free cocoon (lanes 3 and 4) silkworms were subjected to SDS-PAGE, and the resulting gels were stained with Coomassie Brilliant Blue. Bands corresponding to Ser1, FibH, and FibL proteins are indicated by arrows on the right side. The band with the expected reported molecular weight of Ser3 is indicated by an asterisk (*) [S1]. Molecular mass of the marker proteins (in kDa) is indicated on the left.

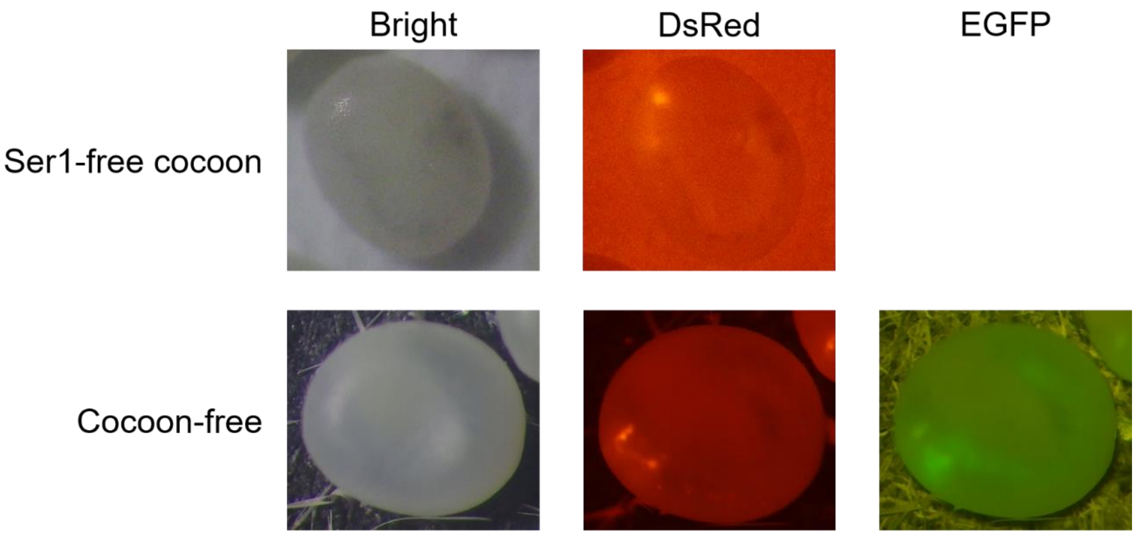

Figure S3. Bright field and fluorescent images of Ser1-free cocoon silkworms (upper panel), and cocoon-free silkworms (lower panel) generated from the cross between Ser1-free cocoon and fibroin-free cocoon silkworms at the late embryonic stage. Related to STAR Methods.

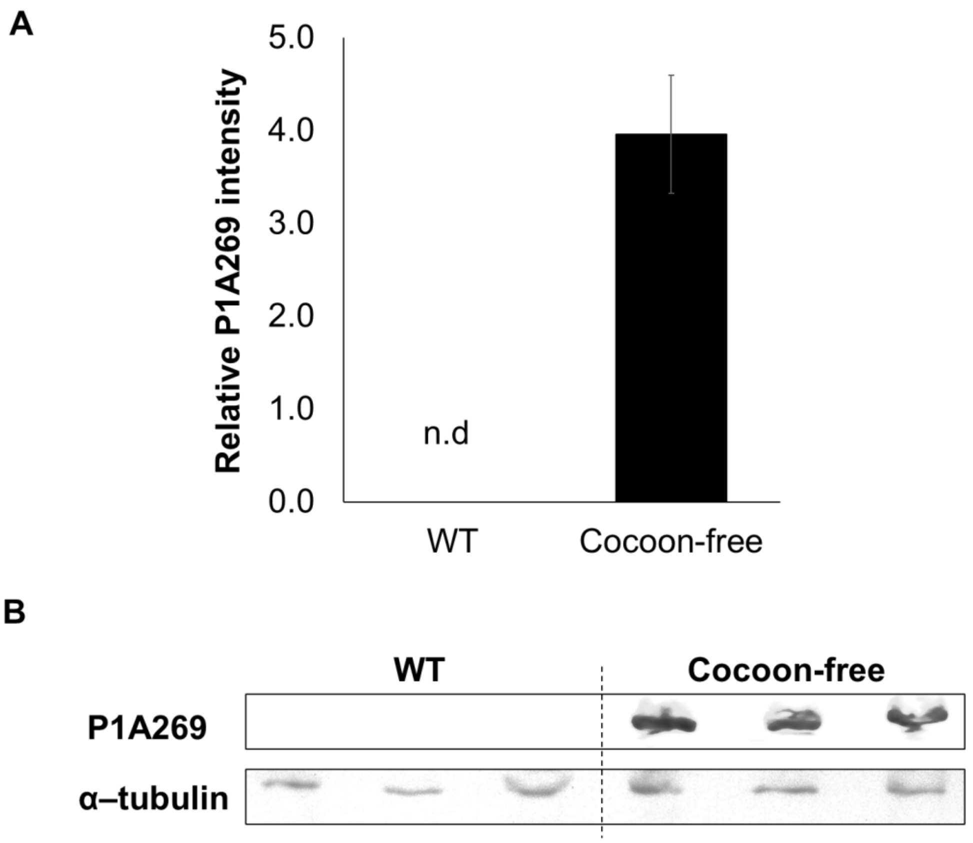

Figure S4. Analysis of the P1A269 expression in the middle silk glands (MSGs) of the cocoon-free silkworm larvae. Related to STAR Methods.
(A and B) P1A269 expressed in the MSGs of cocoon-free silkworm larvae was detected by immunoblot with HRP-conjugated anti-FLAG antibody. The densitometry of P1A269 was normalized to that of α-tubulin. Data represent mean ± SEM (n = 3); n.d., not detected.

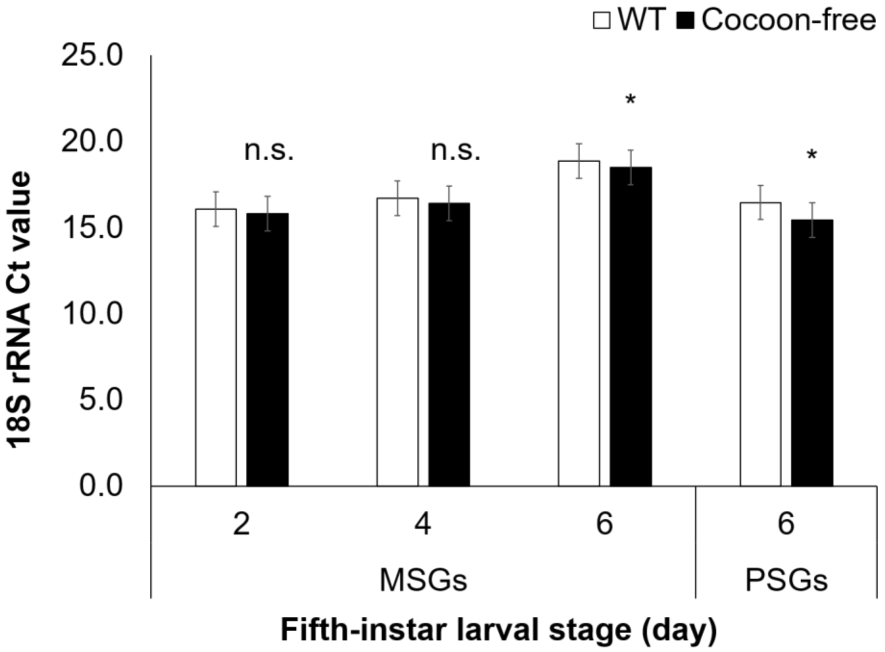

Figure S5. Quantitative RT-PCR analysis of 18s rRNA gene in the MSGs and PSGs of WT and cocoon-free silkworm larvae. Related to STAR methods.
The expression of 18s rRNA in the MSGs and PSGs of day 2, 4 and/or 6 fifth-instar WT and cocoon-free silkworm larvae were analyzed by qRT-PCR and documented as cycle threshold (Ct) values. Data represent mean ± SEM (n = 3); * $p < 0.05$; n.s., not significant, according to the Student's t-test. MSGs, middle silk glands; PSGs, posterior silk glands.

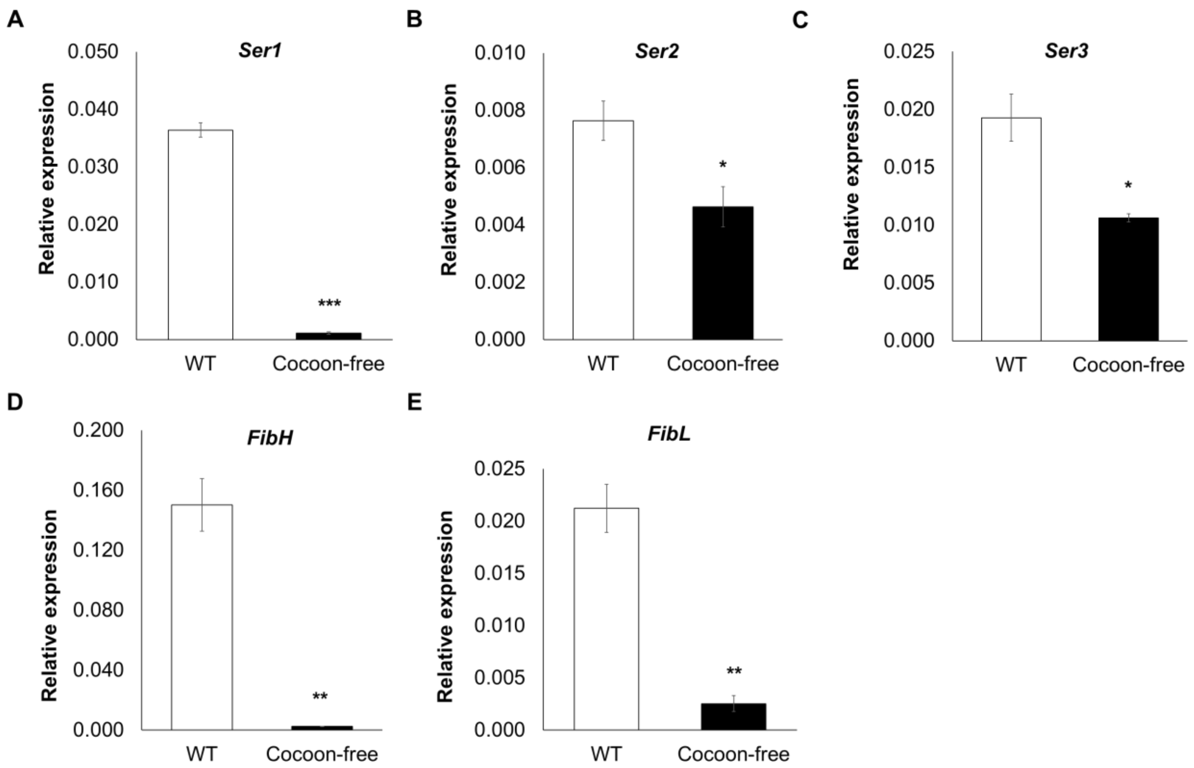

Figure S6. Sericin and fibroin gene expression in the silk glands of WT and cocoon-free silkworm larvae. Related to Figure 1.

(A, B, and C) The mRNA levels of *Ser1*, *Ser2*, and *Ser3* in middle silk glands (MSGs) of the fifth instar larvae at days when their levels appeared to peak, which were at days 4, 2, and 6 respectively.

(D and E) The mRNA levels of *FibH* and *FibL* in the posterior silk glands (PSGs) at day 6 of the fifth instar larvae. The mRNA levels were normalized to 18S rRNA and presented as the relative expression levels. Data represent mean ± SEM (n = 3); * $p < 0.05$; ** $p < 0.01$; *** $p < 0.001$ according to the Student's t-test.

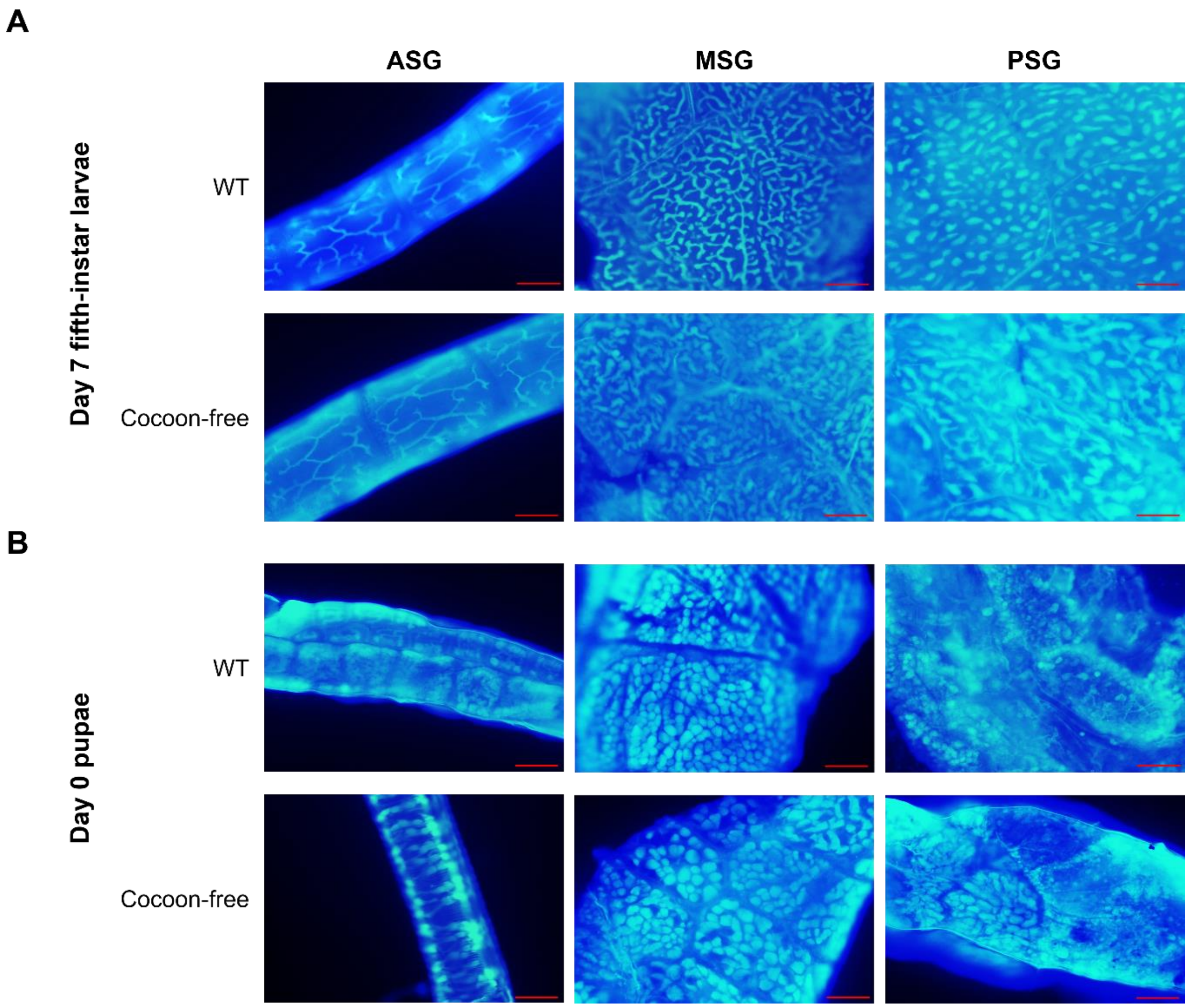

Figure S7. Fluorescence microscopy images of the anterior (ASG), middle (MSG) and posterior (PSG) silk glands dissected from (A) day 7 fifth instar larvae and (B) day 0 pupae of WT and cocoon-free silkworms. Related to Figure 1.

ASG, anterior silk gland; MSG, middle silk gland; PSG, posterior silk gland. Nuclear DNA was stained with DAPI and visualized under a fluorescent microscope (Scale bar, 100µm).

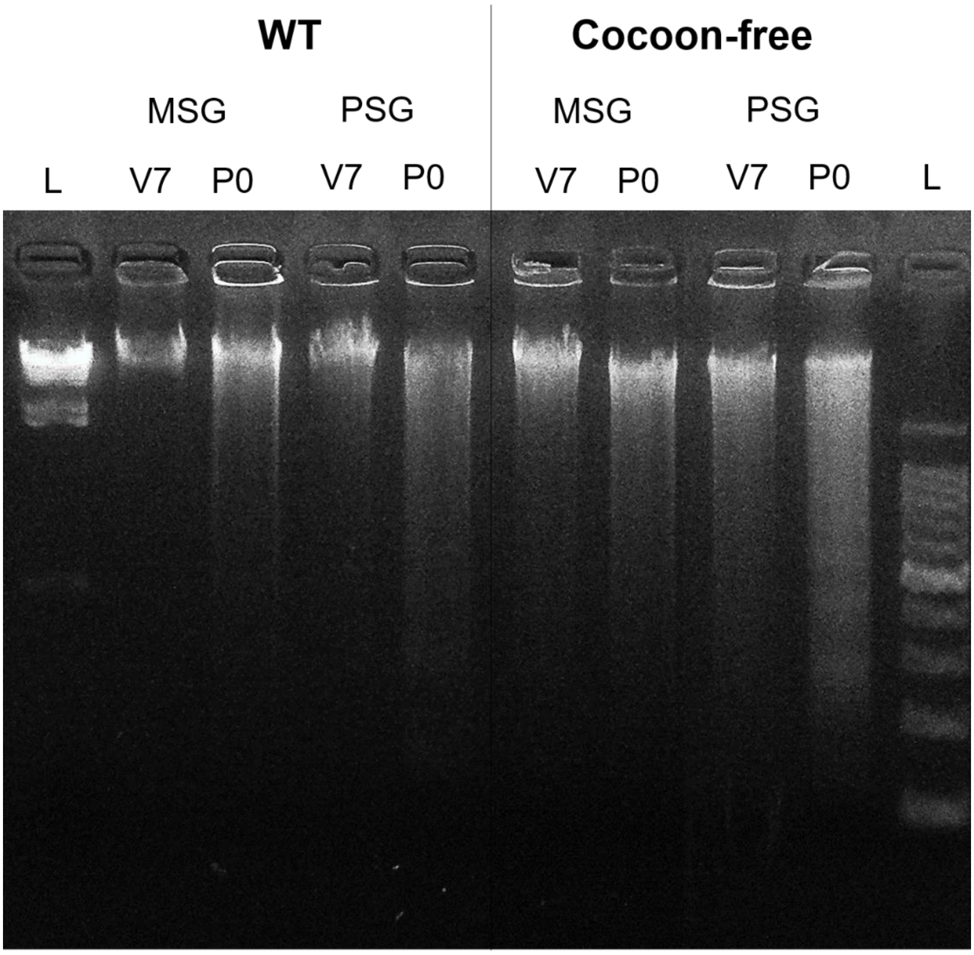

Figure S8. Analysis for DNA fragmentation in the middle (MSG) and posterior (PSG) silk glands of WT and cocoon-free silkworms. Related to Figure 1.
Genomic DNA was extracted from MSGs and PSGs of day 7 fifth instar larvae (V7) and day 0 pupae (P0) of WT and cocoon-free silkworms and electrophoresed on 2% agarose gel. L, DNA ladder.

(A)

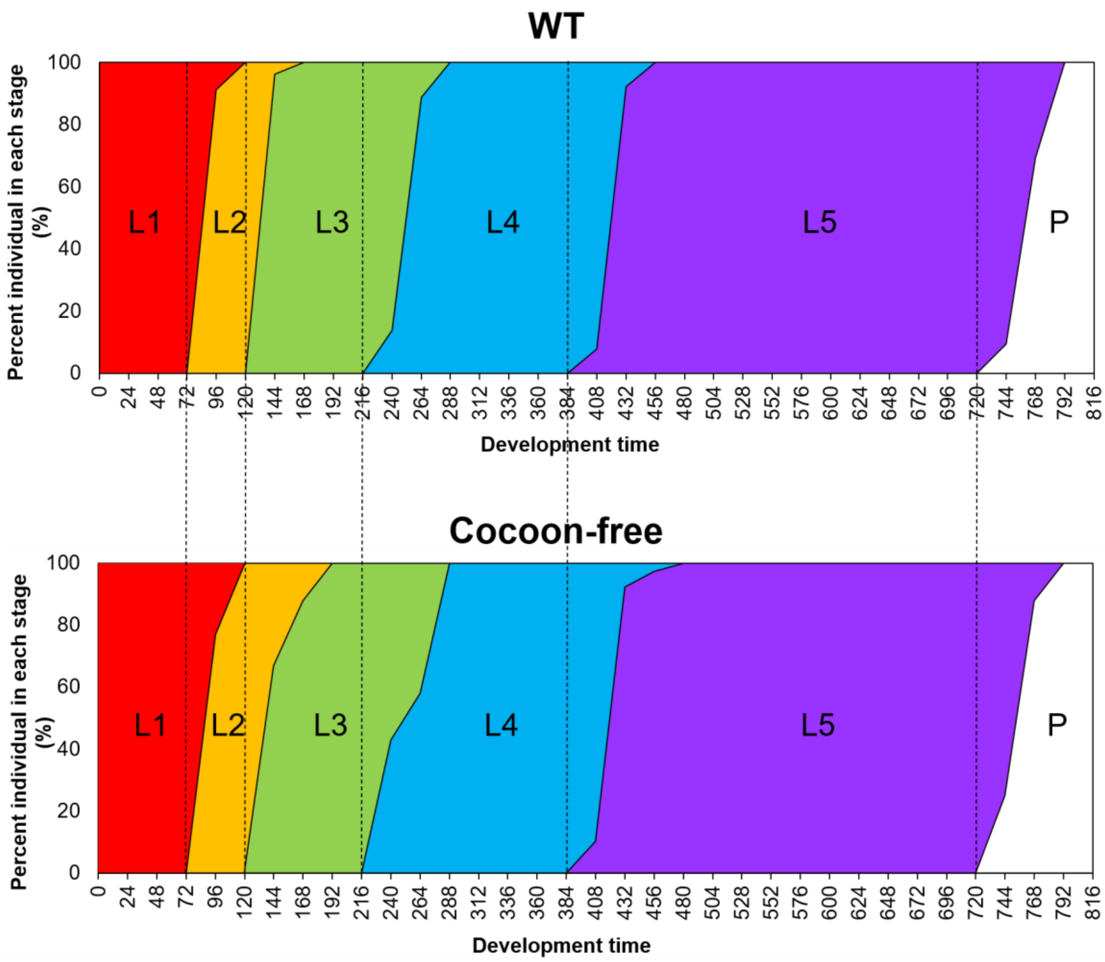

Figure S9. Developmental duration of WT (A) and cocoon-free (B) silkworms from larvae to pupae. Related to Figure 1.

The percentage of individuals (n = 60) and the time points of transition from each larval instar to pupal stages are represented by the curves and dashed lines, respectively. L1, first instar (red); L2, second instar (orange); L3, third instar (green); L4, fourth instar (blue); L5, fifth instar (purple); P, pupae (white).

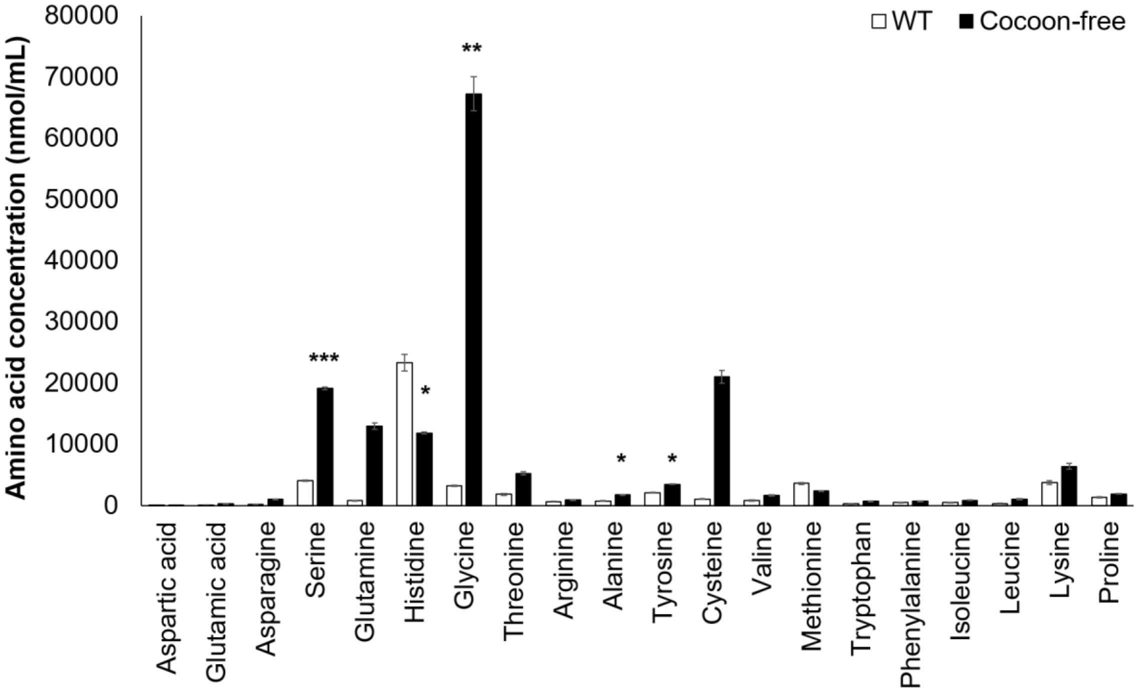

Figure S10. Composition of the major silk amino acid constituents in the larval hemolymph of WT and cocoon-free silkworms. Related to Table 1.

Hemolymph samples (100 µL) were collected from the female WT and cocoon-free silkworm larvae at day 2 after gut purge and ethanol-precipitated before being analyzed by HPLC. The concentrations (in nmol/mL) of amino acids in larval hemolymph are presented. Data represent mean ± SEM (n = 3); * $p < 0.05$; ** $p < 0.01$; *** $p < 0.001$ according to the Student's t-test.

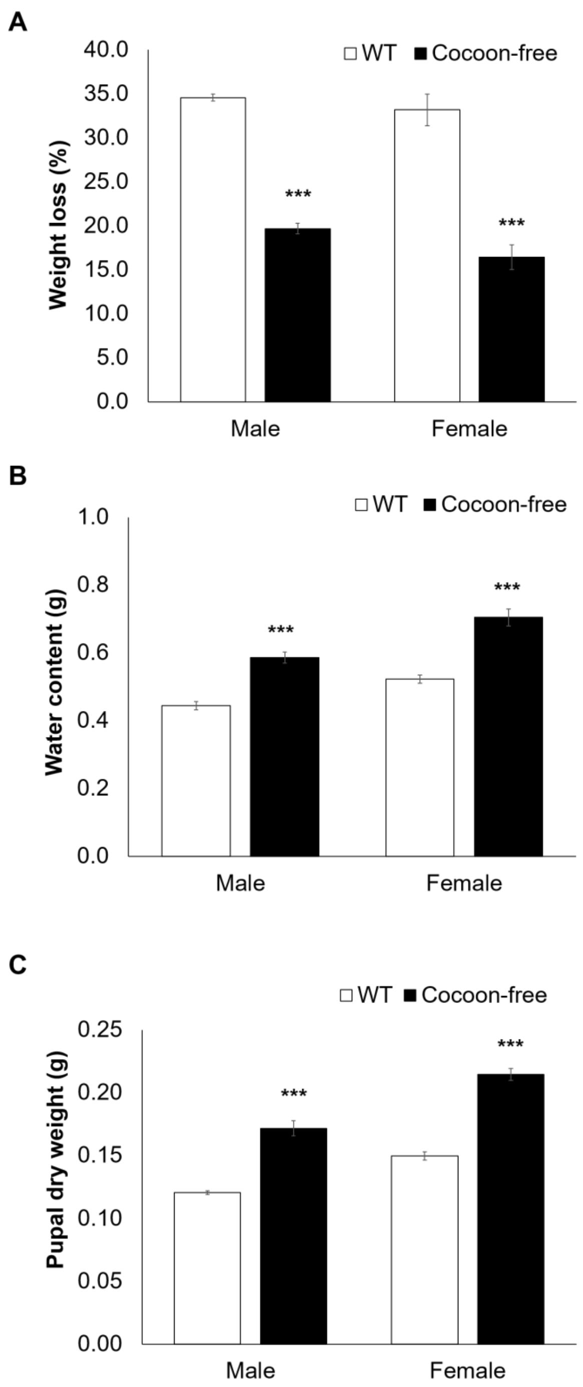

Figure S11. Effect of repressing silk protein synthesis on the weight reduction upon larval-pupal metamorphosis, water content, and pupal dry weight in the cocoon-free silkworms. Related to Figure 2.

(A) Percent weight loss in both male and female WT and cocoon-free silkworms during the larval-pupal metamorphosis. Data are shown as mean ± SEM (n = 15-16 for each sex); *** $p < 0.001$ according to the Student's t-test.

(B) Comparison of water content and (C) pupal dry weight between the male and female WT and cocoon-free silkworm pupae (n = 5 for each sex). Data represent mean ± SEM; *** $p < 0.001$ according to the Student's t-test.

A

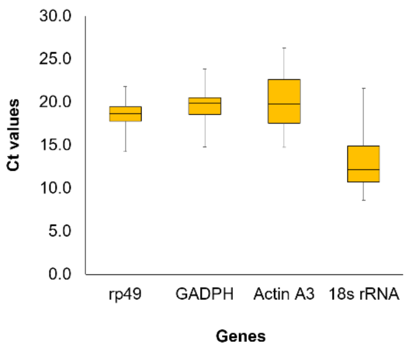

B

Gene	Mean	SD	Min Ct	Max Ct	CV (%)
rp49	18.5	1.8	14.3	21.8	9.6
GADPH	19.6	2.1	14.8	23.9	10.9
Actin A3	20.2	3.2	14.8	26.3	16.0
18s rRNA	13.1	3.3	8.6	21.6	24.8

C

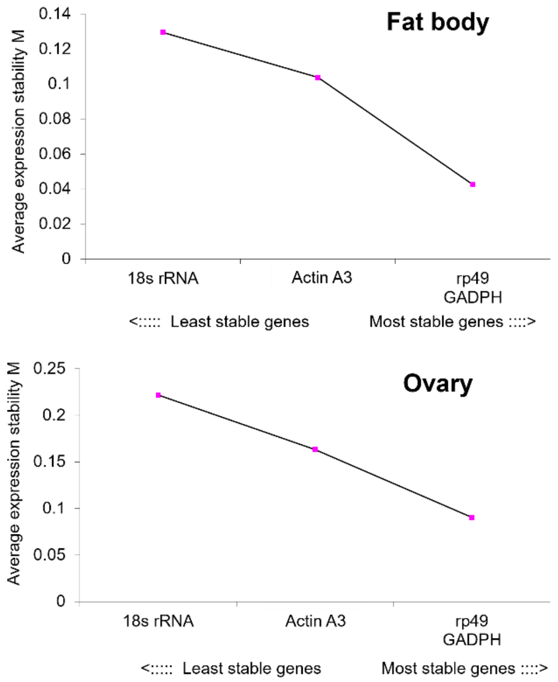

Figure S12. Expression stability of the candidate reference genes in WT and cocoon-free silkworms. Related to STAR Methods.

(A) The expression of *rp49*, *GADPH*, *actin A3* and *18s rRNA* genes across diverse tissues during different pupal stages of WT and cocoon-free silkworms were depicted using cycle threshold (Ct) values, presented as whisker box plots. The box represents the 25th and 75th percentiles, with the line depicting the median and whiskers on each box representing the minimum and maximum Ct values. (B) Expression stability of candidate reference genes across various tissues of different pupal stages between WT and cocoon-free silkworms was assessed by BestKeeper. The stability of genes was established from most stable to least stable, with lower to higher standard deviation (SD) and the coefficient of variation (CV) serving as the criteria. (C) Average expression stability values (M) of candidate reference genes in the ovary or fat body of WT and cocoon-free silkworm pupae were analyzed using geNorm and represented from the least stable (left) to the most stable (right).

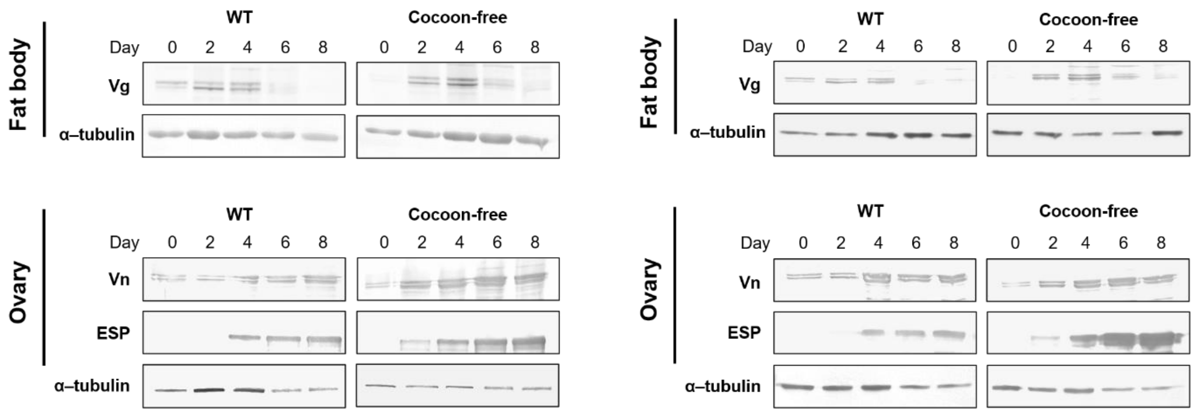

Figure S13. Immunoblot images from additional experiments conducted to investigate the egg yolk protein expression in fat bodies and ovaries of female WT and cocoon-free silkworm pupae from day 0–8 after pupation. Related to Figure 4.

Immunoblot was performed in triplicate, with each involving biologically independent samples (*n* = 3). Equal amounts of total soluble proteins (3 µg) were loaded, probed with specific anti-sera and visualized using DAB substrate. Vg, vitellogenin; Vn, vitellin; ESP, egg-specific protein.

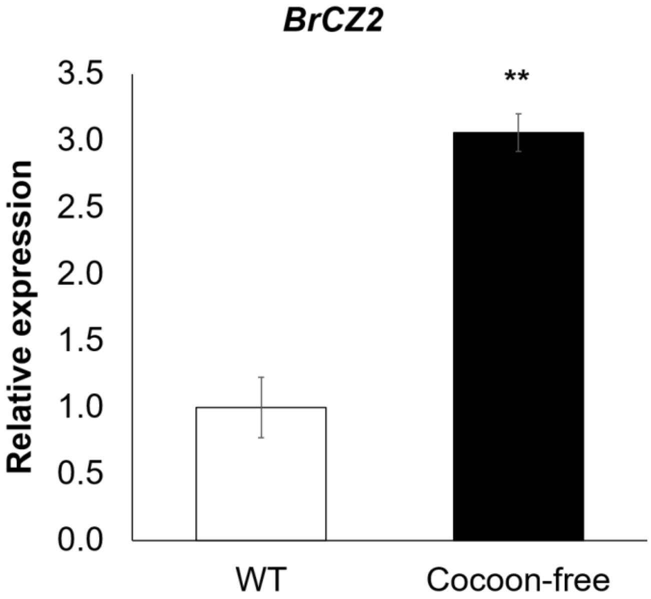

Figure S14. The gene expression level of the transcription factor *BrcZ2* was significantly higher in the pupal fat body of the female cocoon-free silkworms compared to that of WT silkworms. Related to Figure 4.

The day 0 pupal fat body samples from female WT and cocoon-free silkworms were used for qRT-PCR analysis. The *BrcZ2* mRNA levels were normalized to *rp49* and presented as fold-change relative to the WT (set as 1). Data represent mean ± SEM (*n* = 3); ** $p < 0.01$ according to the Student's *t*-test.

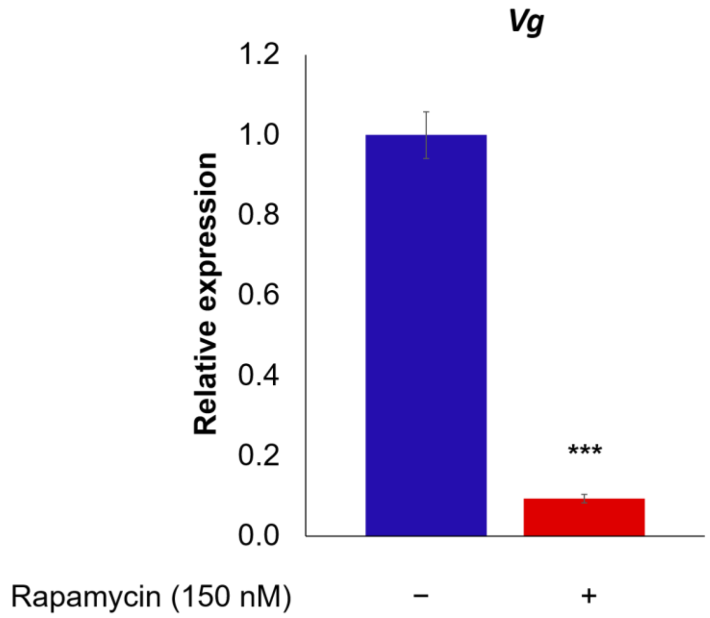

Figure S15. Rapamycin effectively inhibited TOR-mediated *Vg* transcription in cultured cocoon-free fat bodies. Related to Figure 5.

The expression levels of *Vg* mRNA in the cocoon-free silkworm fat bodies (50 mg) cultured with or without rapamycin (150 nM) were analyzed by qRT-PCR. The *Vg* mRNA levels were normalized to *rp49* and presented as fold-change relative to rapamycin untreated group (set as 1). Data represent mean ± SEM (n = 3), *** $p < 0.001$ according to the Student's t-test.

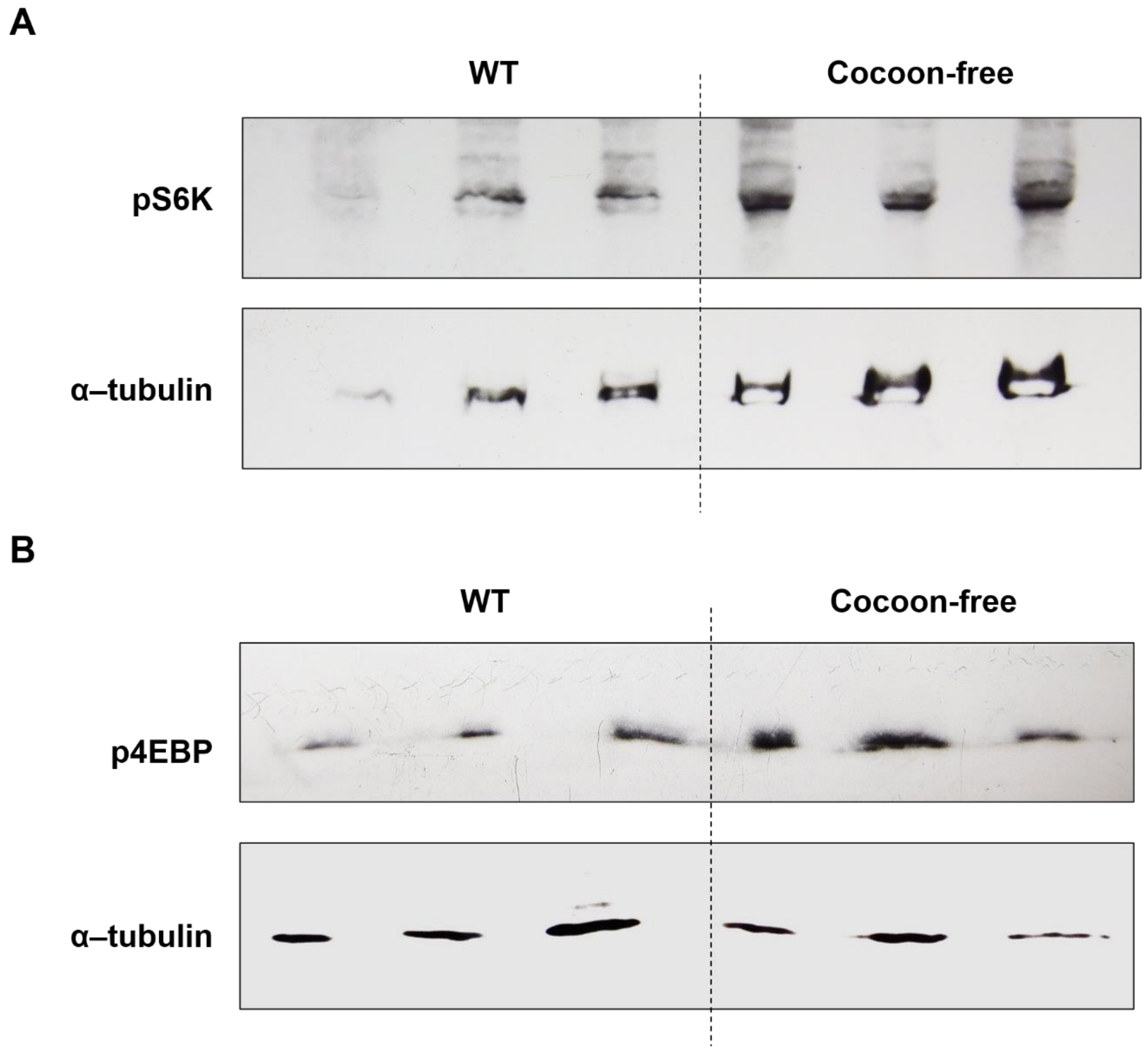

Figure S16. Upregulation of TOR signaling stimulated phosphorylation of (A) S6K and (B) 4EBP in the pupal fat bodies of female cocoon-free silkworms compared to the female WT silkworms. Related to Figure 5.

Immunoblot analysis of phosphorylated S6K (pS6K) and 4EBP (p4EBP) expression in the pupal fat bodies of female WT and cocoon-free silkworms. Equal amounts of total soluble proteins (100 µg) were loaded, probed with phospho-specific antibodies and visualized with Chemi-Lumi One L. Densitometry of the immunoreactive bands were normalized to that of α-tubulin and data are shown in Figure 5. Each lane represents one independent individual from each group.

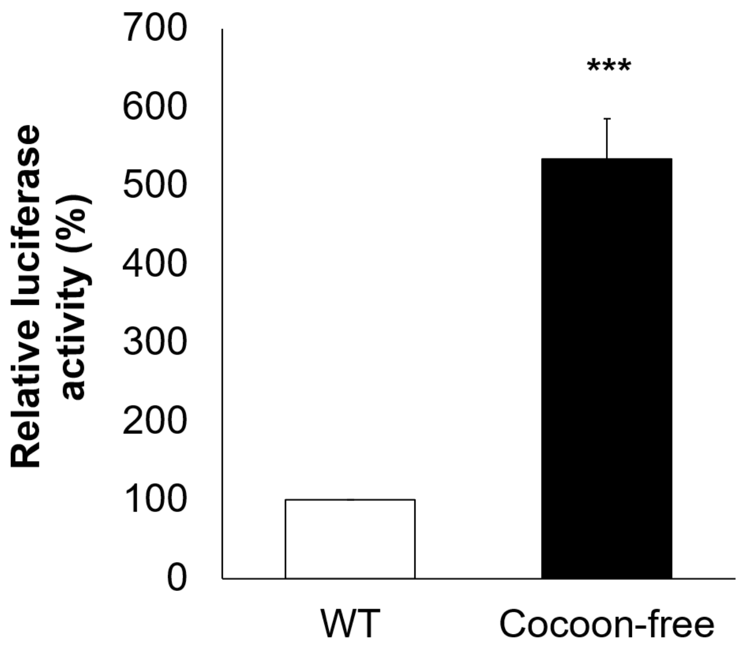

Figure S17. Luciferase expression in the female cocoon-free silkworm pupae was significantly higher than that of the WT silkworms. Related to Figure 5.
BmNPV-luciferase virus was diluted to 2 × 10^{13} pfu/mL and 50 µL was injected into each pupa. Each infected pupa was homogenized in 1× PBS, further diluted 5000×, and assayed for luciferase activity using the PicaGene BrillianStar-LT luminescence kit. The relative luminescence unit (RLU) for the female cocoon-free silkworm pupae was expressed as a percentage of the WT (set at 100%). Data represent mean ± SEM (n = 5); *** $p < 0.001$ according to the Student's t-test.

Table S1. Oligonucleotide primers used in this study. Related to STAR Methods.

Primer names	Primer sequences (5'→3')	Use
(For cloning)		(Vector constructs)
Ser1fKpnI	TTTGGTACCAGCGGTCAGAAACCTTGTTAACC	pIZ-Ser1Pro-H1/P1A269/FLAG
Ser1rBamHI	TTTGGATCCGTTGGCGGTCTTTGGATCGCTTG	
Ser1-P1Af	GAACATTGTCAGATCTGCGGAGGGACTTTAGTTCTCTCT	pBacMCS[Ser1Pro-P1A269, 3 × P3-DsRed] vector
Ser1-P1Ar	GGTTTGTCCAAACTCTCCCCTGATTCTGTGGATAACCG	
(For inverse PCR)		
UAS RF2	GCGTGAGTCAAAATGACGCATGAT	pBacMCS[Ser1Pro-P1A269, 3 × P3-DsRed] vector
UAS RR2	ATCAGTGACACTTACCGCATTGACA	
UAS LF1	CCTCGATATACAGACCGATAAAACAC	
UAS LR2	AACTTTTATGGCGCGCCATCGAAT	
UAS LR1	GACTGAGATGTCCTAAATGCACAG	
UAS RF3	GACCGATAAACACATGCG	
(For qRT-PCR)		(Purpose)
18S rRNA-1	CGATCCGCCGACGTTACTACA	Silk gland gene expression analysis
18S rRNA-2	GTCCGGGszCCTGGTGAGATTT	
Ser1-1	CAAAGACCGCCAACATGCGT	
Ser1-2	CACTGCTAGCTGCATTGTACTTTCG	
Ser2-1	CGGCTGACTACCAAACCAAA	
Ser2-2	CCGAGAGTTGCTGCCCTTAC	
Ser3-1	CCAGAGCCAGTCATACAACAAAG	
Ser3-2	TGTCGTCGGAATTCTACACCA	
FibH-1	TCCGACGGTAACGAGTCCATTG	
FibH-2	TACGTATGGCCCGCTCTGAGAA	
FibL-1	CGTCATCAACCCTGGTCAAC	
FibL-2	GCGGCTTCGAAGTCATAGAT	
Vg-1	GCCTCGATTTTCCAACTTCA	Reproduction gene expression analysis
Vg-2	CCATTCTGAAGCAACAGGAG	
VgR-1	GAGTGCCTGGGCGAGGATGT	
VgR-2	CTGAGCGTCTGGCTTGTGA	
ESP-1	TTTGACTGTCCTGCGCATTC	
ESP-2	ATTACCCAGCCATACGTCGT	
Bmovo1-1	CAGTACTTACCCGTTTTGGT	
Bmovo1-2	CCGTTTGCAATTTTAGAACTGC	
rp49-1	CAGGCGGTTCAAGGGTCAATAC	Nutritional and hormonal signaling gene expression analysis
rp49-2	TGCTGGGCTCTTTCCACGA	
InR-1	CCGAACTAGAAGTGTCCCAAGA	
InR-2	CCACTGCGATTTGCGTTAGC	
Akt-1	TCCCGCCGTAGATTGGT	
Akt-2	GATGAGCCCGAACAGCAC	
TOR-1	GTGAACTTTATCATTATGCGAAGACGG	
TOR-2	CCGATTAGGCATACAATAGCAAG	
FOXO-1	GGTTACCAGAGCCCTTGGTC	
FOXO-2	TTGGAAGCGGCCACGTGTTG	
4EBP-1	ATGTTATCCCTTCGGCAATCTC	
4EBP-2	GCTGAAGGTTTCCTGCGACTC	
BrcZ2-1	ATGGTGGACAGTCAGACGCAACA	
BrcZ2-2	AGCGTCATTGTGTGTGAGACC	

Table S2. Genomic location of the transgene in the Ser1-free cocoon line generated by microinjection with the pBacMCS[Ser1Pro-P1A269, 3 × P3-DsRed] plasmid. Related to STAR Methods.

Silkworm line	Sequence at the border of the transgene[a]	Chromosome number
Ser1-free cocoon	GTTAGGTGCC**TTAA**(transgene)**TTAA**CTA GGATTTCccz	Chromosome 19

[a] Transgene bordered by the characteristic TATA recognition motifs of piggyBac, as indicated in bold, was detected by inverse PCR and blast searches against silkworm genome database via KAIKOBLAST (https://sgp.dna.affrc.go.jp/KAIKObase/) to identify transgene insertion site.

References

S1. Takasu, Y., Hata, T., Uchino, K., and Zhang, Q. (2010). Identification of Ser2 proteins as major sericin components in the non-cocoon silk of Bombyx mori. Insect Biochem. Mol. Biol. *40*, 339–344. 10.1016/j.ibmb.2010.02.010.
